# Corrigendum to “Expert management of congenital portosystemic shunts and their complications” [JHEP Reports 6 (2024)]

**DOI:** 10.1016/j.jhepr.2024.101024

**Published:** 2024-01-30

**Authors:** Valérie Anne McLin, Stéphanie Franchi-Abella, Timothée Brütsch, Atessa Bahadori, Valeria Casotti, Jean de Ville de Goyet, Grégoire Dumery, Emmanuel Gonzales, Florent Guérin, Sebastien Hascoet, Nigel Heaton, Béatrice Kuhlmann, Frédéric Lador, Virginie Lambert, Paolo Marra, Dr Aurélie Plessier, Alberto Quaglia, Anne-Laure Rougemont, Laurent Savale, Moinak Sen Sarma, Olivier Sitbon, Riccardo Antonio Superina, Hajime Uchida, Mirjam van Albada, Hubert Petrus Johannes van der Doef, Valérie Vilgrain, Julie Wacker, Nitash Zwaveling-Soonawala, Dominique Debray, Barbara Elisabeth Wildhaber

**Affiliations:** 1 Swiss Pediatric Liver Center, Gastroenterology, hepatology and pediatric nutrition unit, Department of pediatrics, Gynecology and Obstetrics, University of Geneva, Geneva, Switzerland; 2 Université Paris-Saclay, Faculté de médecine, Le Kremlin-Bicêtre, France; 3 AP-HP, Centre de référence des maladies rares du foie de l’enfant, Service de radiologie pédiatrique diagnostique et interventionnelle, Hôpital Bicêtre, Le Kremlin-Bicêtre, France; 4 BIOMAPS UMR 9011 CNRS, INSERM, CEA, Orsay, France; 5 ERN RARE LIVER; 6 ERN Transplant Child; 7 Communication in Science, rue du Tunnel 7, Carouge, Switzerland; 8 Department of pediatrics, Gynecology and Obstetrics, University of Geneva, Geneva, Switzerland; 9 Pediatric Hepatology, Gastroenterology and Transplant Centre, ASST Papa Giovanni XXIII Hospital, Bergamo, Italy; 10 Pediatric Department for the Treatment and Study of Abdominal Diseases and Abdominal Transplantation, ISMETT UPMC, Palermo, Italy; 11 AP-HP, Service de gynécologie et d’obstétrique, Hôpital Bicêtre, Le Kremlin-Bicêtre, France; 12 AP-HP, Centre de référence des maladies rares du foie de l’enfant, FHU Hepatinov, Service d’hépatologie et transplantation hépatique pédiatriques, Hôpital Bicêtre, Le Kremlin-Bicêtre, France; 13 INSERM UMRS_1193, Orsay, France; 14 AP-HP, Service de chirurgie pédiatrique, Hôpital Bicêtre, Le Kremlin-Bicêtre, France; 15 Department of Congenital Heart Diseases, Hôpital Marie Lannelongue, France; 16 INSERM UMR_S 999, Université Paris, France; 17 Institute of Liver Studies, Kings College Hospital, London, England; 18 Pediatric Endocrinology, Cantonal Hospital Aarau KSA, Aarau, Switzerland; 19 Service de Pneumologie, University of Geneva, Geneva, Switzerland; 20 Cardiologie congénitale, Institut Mutualiste Montsouris, Paris, France; 21 Department of Radiology, Papa Giovanni XXIII Hospital, School of Medicine and Surgery - University of Milano-Bicocca, Bergamo, Italy; 22 Centre de référence des maladies vasculaires du foie, Service d’hépatologie Hôpital Beaujon, Clichy, France; 23 VALDIG; 24 Department of Cellular Pathology, Royal Free London NHS Foundation Trust/UCL Cancer Institute, London, England; 25 Swiss Pediatric Liver Center, Division of Clinical Pathology, Diagnostic Department, University of Geneva, Geneva, Switzerland; 26 AP-HP, Centre de référence de l’hypertension pulmonaire, Service de pneumologie et soins intensifs respiratoires, Hôpital Bicêtre, Le Kremlin-Bicêtre, France; 27 INSERM UMR_S 999, Hôpital Marie Lannelongue, Le Plessis Robinson, France; 28 ERN Lung; 29 Department of Pediatric Gastroenterology, Sanjay Gandhi Postgraduate Institute of Medical Sciences, Lucknow, India; 30 Northwestern University Feinberg School of Medicine, Ann & Robert H. Lurie Children’s Hospital of Chicago, Chicago, Illinois, USA; 31 Organ Transplantation Center, National Center for Child Health and Development, Tokyo, Japan; 32 Department of paediatric and congenital cardiology, University Medical Center Groningen, University of Groningen, The Netherlands; 33 Division of paediatric gastroenterology and hepatology, Department of paediatrics, University Medical Center Groningen, Groningen, The Netherlands; 34 Université Paris Cité, CRI, INSERM, Paris, France; 35 AP-HP, Département de Radiologie, Hôpital Beaujon. Nord, Clichy, France; 36 Pediatric cardiology unit, Department of pediatrics, gynecology and obstetrics, University of Geneva, Geneva, Switzerland; 37 Centre Universitaire Romand de Cardiologie et Chirurgie Cardiaque Pédiatrique, University of Geneva and Lausanne, Switzerland; 38 Department of Pediatric Endocrinology, Amsterdam University Medical Centers, University of Amsterdam, Amsterdam, The Netherlands; 39 AP-HP, Unité d’hépatologie pédiatrique et transplantation hépatique, Hôpital Necker, Paris, France; 40 Centre de Référence des maladies rares du foie de l’enfant, FILFOIE, France; 41 Swiss pediatric Liver Center, Division of pediatric surgery, Department of pediatrics, gynecology, and obstetrics, University of Geneva, Geneva, Switzerland

It has come to our attention that we did not include the full surname of one of the authors in our publication. ‘Nitash Zwaveling’ should read ‘Nitash Zwaveling-Soonawala’. We apologise for any inconvenience caused.

