# Supplementary material for: Expert management of congenital portosystemic shunts and their complications
Source: JHEP Rep. 2023 Oct 20;6(1):100933. doi: 10.1016/j.jhepr.2023.100933 (PMC10792643; doi:10.1016/j.jhepr.2023.100933)
Supplement: Multimedia component 1 [file mmc1.pdf]

Expert management of congenital portosystemic shunts and their complications

Paolo Marra, Stephanie Franchi-Abella, Timothée Brütsch, Valérie McLin

Table of contents

How to perform an occlusion test………………………………………………………..……..2
Supplementary references……………………………………………………………….. 2

How to perform an occlusion test

The occlusion test aims to simulate the portosystemic shunt closure: to allow the opacification of a hypoplastic and/or ectopic portal system, if present, and to depict its anatomy; to monitor the increase of portal pressure and to rule out the development of portal hypertension or the opening of other neglected portosystemic communications; to plan the radiological embolization of the shunt. To be effective, the occlusion test must avoid the inadvertent closure of ectopic portal vessels that may originate close to the portosystemic communication, while completely occluding the shunt lumen [1]. The procedure can be performed via the transfemoral and/or the transjugular route under sedation or general anaesthesia, according to the clinical context and resources. Since extrahepatic end-to-side and side-to-side shunts may be very large, dedicated occlusion balloons are preferrable. For example, the Equalizer balloon catheter is currently available on-the-market. Large-caliber aortic or valvuloplasty balloons generally have an ellipsoid shape, which may hinder the correct execution of the test. The balloon should be inflated within the inferior vena cava or the systemic afferent vein at the shunt orifice. If the tip of the balloon catheter is navigated within the shunt, the portal pressure can be determined through the main lumen of the balloon catheter after removing the guidewire. If the tip is in the vena cava lumen, a side catheterization of the spleno-mesenteric axis must be performed. It is important not to perform the occlusion of the vena cava at the level of the hepatic vein confluence, to avoid overestimating the portal pressure due to hepatic outflow obstruction. Intrahepatic or extrahepatic shunts originating distant from the spleno-mesenteric confluence may be occluded more selectively, with widely available smaller occlusion balloon catheters. There is no consensus about the duration of the occlusion test. Given that prolonged occlusion of large shunts may cause hemodynamic collapse or thrombosis, it is reasonable to limit occlusion duration just to the time required for wedge portal pressure measurement and portal venography execution. In such circumstances heparin prophylaxis is not recommended. No consensus exists also regarding a cut-off for portal pressure value allowing the choice between one-stage and two-stage shunt closure: an absolute wedge portal pressure >30 mmHg and/or a >20 mmHg gradient between free and wedge portal pressure are commonly adopted criteria to promote a two-stage closure [2,3].

Supplementary references

[1] Franchi-Abella S, Gonzales E, Ackermann O, Branchereau S, Pariente D, Guérin F, et al. Congenital portosystemic shunts: diagnosis and treatment. Abdom Radiol (NY) 2018;43:2023–36. https://doi.org/10.1007/s00261-018-1619-8.

[2] Matsuura T, Takahashi Y, Yanagi Y, Yoshimaru K, Yamamura K, Morihana E, et al. Surgical strategy according to the anatomical types of congenital portosystemic shunts in children. Journal of Pediatric Surgery 2016;51:2099–104. https://doi.org/10.1016/j.jpedsurg.2016.09.046.

[3] Rajeswaran S, Johnston A, Green J, Riaz A, Thornburg B, Mouli S, et al. Abernethy Malformations: Evaluation and Management of Congenital Portosystemic Shunts. J Vasc Interv Radiol 2020;31:788–94. https://doi.org/10.1016/j.jvir.2019.08.007.
