# Supplementary material for: Expert management of congenital portosystemic shunts and their complications
Source: JHEP Rep. 2023 Oct 20;6(1):100933. doi: 10.1016/j.jhepr.2023.100933 (PMC10792643; doi:10.1016/j.jhepr.2023.100933)
Supplement: Multimedia component 4 [file mmc4.pdf]

Expert management of congenital portosystemic shunts and their complications

Valérie Anne McLin,[1,5,*] Stéphanie Franchi-Abella,[2,3,4,5,6] Timothée Brütsch,[7] Atessa Bahadori,[8] Valeria Casotti,[9,6] Jean de Ville de Goyet,[10] Grégoire Dumery,[2,11] Emmanuel Gonzales,[2,12,13,5,6] Florent Guérin,[2,14,5,6] Sebastien Hascoet,[15,16] Nigel Heaton,[17] Béatrice Kuhlmann,[18] Frédéric Lador,[19] Virginie Lambert,[20,3] Paolo Marra,[21] Aurélie Plessier,[22,5,23] Alberto Quaglia,[24] Anne-Laure Rougemont,[25] Laurent Savale,[2,26,27,28] Moinak Sen Sarma,[29] Olivier Sitbon,[2,26,27,28] Riccardo Antonio Superina,[30] Hajime Uchida,[31] Mirjam van Albada,[32] Hubert Petrus Johannes van der Doef,[33] Valérie Vilgrain,[34,35,5,23] Julie Wacker,[36,37] Nitash Zwaveling,[38] Dominique Debray,[39,40,5,6,†] Barbara Elisabeth Wildhaber[41,5,†]

Summary

Congenital portosystemic shunts are often associated with systemic complications, the most challenging of which are liver nodules, pulmonary hypertension, endocrine abnormalities, and neurocognitive dysfunction. In the present paper, we offer expert clinical guidance on the management of liver nodules, pulmonary hypertension, and endocrine abnormalities, and we make recommendations regarding shunt closure and follow-up.

© 2023 The Author(s). Published by Elsevier B.V. on behalf of European Association for the Study of the Liver (EASL). This is an open access article under the CC BY license (http://creativecommons.org/licenses/by/4.0/).

Keywords: congenital portosystemic shunt; pulmonary hypertension; hepatocellular carcinoma; adenoma; focal nodular hyperplasia; β-catenin; hyperandrogenism; puberty; hypoglycemia; hyperinsulinism; occlusion test; portal pressure

Received 9 May 2023; received in revised form 18 September 2023; accepted 25 September 2023; available online 20 October 2023

Introduction

Congenital portosystemic shunt(s) (CPSS) are rare vascular malformations of embryonic origin through which intestinal blood flow bypasses the liver partially or completely, and thereby reaches the systemic circulation unfiltered (Fig. 1). The shunt can occur within the liver (intrahepatic CPSS) or outside the liver (extrahepatic CPSS).

CPSS are diagnosed by Doppler ultrasound or cross-sectional imaging in a patient presenting with signs or symptoms characteristic of CPSS, such as liver nodules, pulmonary vascular disease, endocrine abnormalities, neurocognitive symptoms, or increasingly, as part of the workup of congenital heart disease (CHD) or syndromes. The gold standard to confirm the diagnosis and analyse anatomy is a phlebography with an occlusion test.

Potential clinical complications associated with CPSS are numerous (summarised in Table 1). Although increasingly recognised, the management of some of these complications is challenging and controversial even amongst experienced centres. In addition, CPSS have been reported in numerous syndromes or congenital malformations for which the reader is referred to another report.[1] In July of 2022, experts and members of the IRCPSS (International Registry of Congenital Portosystemic Shunts) met to discuss these issues, with the aim of reaching a consensus on some critical aspects of management and outlining priorities for the generation of evidence. Indeed, owing to the rarity of this malformation, evidence is still scarce, and data from the registry is forthcoming. Therefore, this position paper aims to offer a carefully discussed and thoughtful expert opinion on the management of these complex patients, who should undergo comprehensive head-to-toe assessment Fig. 2, management, and follow-up once referred to expert centres. In addition, we provide recommendations on the timing and method of shunt closure, pre-closure workup, and long-term follow-up.

Methods

The moderators and speakers of the symposium were invited by the lead authors to draft their recommendations based on their review of the literature, presentations, and discussions. Given the multidisciplinary nature of this effort, the expert recommendations presented herein were agreed upon consensually within each subspecialty (interventional radiology, radiology, hepatology, surgery, endocrinology, obstetrics, histopathology, cardiology, and pulmonary medicine).

Approach to the patient with CPSS and liver nodules

Patients with CPSS have a high cumulative prevalence of liver masses, reaching 73% (Table 1). The management of liver nodules in patients with CPSS is challenging for several reasons. Their radiological

[1]Swiss Pediatric Liver Center, Gastroenterology, Hepatology and Pediatric Nutrition Unit, Department of pediatrics, Gynecology and Obstetrics, University of Geneva, Geneva, Switzerland; [2]Université Paris-Saclay, Faculté de médecine, Le Kremlin-Bicêtre, France; [3]AP-HP, Centre de référence des maladies rares du foie de l'enfant, Service de radiologie pédiatrique diagnostique et interventionnelle, Hôpital Bicêtre, Le Kremlin-Bicêtre, France; [4]BIOMAPS UMR 9011 CNRS, INSERM, CEA, Orsay, France; [5]ERN RARE LIVER; [6]ERN Transplant Child; [7]Communication in Science, rue du Tunnel 7, Carouge, Switzerland; [8]Department of pediatrics, Gynecology and Obstetrics, University of Geneva, Geneva, Switzerland; [9]Pediatric Hepatology, Gastroenterology and Transplant Centre, ASST Papa Giovanni XXIII Hospital, Bergamo, Italy; [10]Pediatric Department for the Treatment and Study of Abdominal Diseases and Abdominal Transplantation, ISMETT UPMC, Palermo, Italy; [11]AP-HP, Service de gynécologie et d'obstétrique, Hôpital Bicêtre,

ELSEVIER

Le Kremlin-Bicêtre, France; [12]AP-HP, Centre de référence des maladies rares du foie de l'enfant, FHU Hepatinov, Service d'hépatologie et transplantation hépatique pédiatriques, Hôpital Bicêtre, Le Kremlin-Bicêtre, France; [13]INSERM UMRS_1193, Orsay, France; [14]AP-HP, Service de chirurgie pédiatrique, Hôpital Bicêtre, Le Kremlin-Bicêtre, France; [15]Department of Congenital Heart Diseases, Hôpital Marie Lannelongue, France; [16]INSERM UMR_S 999, Université Paris, France; [17]Institute of Liver Studies, Kings College Hospital, London, England; [18]Pediatric Endocrinology, Cantonal Hospital Aarau KSA, Aarau, Switzerland; [19]Service de Pneumologie, University of Geneva, Geneva, Switzerland; [20]Cardiologie congénitale, Institut Mutualiste Montsouris, Paris, France; [21]Department of Radiology, Papa Giovanni XXIII Hospital, School of Medicine and Surgery - University of Milano-Bicocca, Bergamo, Italy; [22]Centre de référence des maladies vasculaires du foie, Service d'hépatologie Hôpital Beaujon, Clichy, France; [23]VALDIG; [24]Department of Cellular Pathology, Royal Free London NHS Foundation Trust/ UCL Cancer Institute, London, England; [25]Swiss Pediatric Liver Center, Division of Clinical Pathology, Diagnostic Department, University of Geneva, Geneva, Switzerland; [26]AP-HP, Centre de référence de l'hypertension pulmonaire, Service de pneumologie et soins intensifs respiratoires, Hôpital Bicêtre, Le Kremlin-Bicêtre, France; [27]INSERM UMR_S 999, Hôpital Marie Lannelongue, Le Plessis Robinson, France; [28]ERN Lung; [29]Department of Pediatric Gastroenterology, Sanjay Gandhi Postgraduate Institute of Medical Sciences, Lucknow, India; [30]Northwestern University Feinberg School of Medicine, Ann & Robert H. Lurie Children's Hospital of Chicago, Chicago, Illinois, USA; [31]Organ Transplantation Center, National Center for Child Health and Development, Tokyo, Japan; [32]Department of paediatric and congenital cardiology, University Medical Center Groningen, University of Groningen, The Netherlands; [33]Division of paediatric gastroenterology and hepatology, Department of paediatrics, University Medical Center Groningen, Groningen, The Netherlands;

characteristics, molecular profiles, and risk of malignant degeneration differ from those seen in other vascular liver disorders. Moreover, several types of lesions may arise either sequentially or concurrently in the same individual, with a complex identification, as radiological diagnosis sometimes differs from final pathological diagnosis.

Most liver nodules are benign, such as focal nodular hyperplasia (FNH) and hepatocellular adenoma (HCA), and tend to occur at a younger age than hepatocellular carcinoma (HCC),[5,14] which is more frequently reported in teenagers and adults. That said, both HCC and hepatoblastoma (HB) have been described in children with CPSS.[6,15,16] Size seems to increase progressively from benign FNH and nodular regenerative hyperplasia (NRH), to HCA and HCC.[5,14] Finally, the anatomic form of CPSS may play a role in tumour type, owing to the degree of portal flow and compensatory arterial supply. Intrahepatic CPSS are typically associated with benign liver masses, while both benign and malignant tumours have been reported in extrahepatic CPSS.[5,6,17]

Any patient with a new CPSS diagnosis needs a workup for liver nodules. As liver lesions of varying types may co-exist, we recommend initially characterising *each* nodule, in so far as this is feasible, and then monitoring *all* nodules longitudinally. By 'characterise', we mean to define beyond reasonable doubt the nature of each nodule, using imaging and histopathology as required, repeatedly when necessary. Conversely, CPSS should always be looked for in patients, especially younger ones, presenting with liver nodules without clear signs – or a history – of liver disease.

Diagnostic imaging and follow-up prior to shunt closure

Liver imaging is challenging in patients with CPSS because of portal deprivation and increased arterial supply. Contrast-enhanced MRI and contrast-enhanced ultrasound are the preferred methods to image nodules as they best capture mild hyperenhancement. MRI with hepatobiliary contrast agents is essential for baseline evaluation, as lesion signal intensity on hepatobiliary sequences is key to characterising liver nodules in CPSS. Longitudinal imaging can be performed using MRI with extracellular contrast agents, but if concerning features are detected at any point during follow-up, reverting to hepatobiliary agents is preferred to improve nodule characterisation.

Careful and appropriate serial imaging is essential because of the idiosyncrasies of liver nodules in CPSS. For one, although most nodules are benign hepatocellular lesions such as FNH and HCA,[5,14,18] the risk of malignant transformation is real, yet difficult to quantify. Next, FNH may deviate from standard FNH criteria,[19] and for this reason these lesions are sometimes referred to as "FNH-like lesions". Here we have decided to use the term "FNH", while keeping in mind the potential for unusual radiological, histological, and molecular findings. FNH lesions in this context frequently show no central scar, and only weak hyperenhancement on hepatic arterial phase, because high non-nodular liver enhancement results in diminished nodule-to-liver contrast ratio. Finally, HCA subtyping on imaging is also very difficult in the setting of CPSS.

Before shunt closure, imaging is recommended every 6 months, using the preferred imaging modalities described above and depending on local experience and availability. When in doubt about the nature of a nodule, imaging should be repeated every 3 months, or a biopsy obtained for histopathological diagnosis (see next section). Likewise, hepatocellular lesions with evidence of β-catenin activation (see below) also require quarterly imaging using age-appropriate methods. In any case, we suggest performing contrast-enhanced MRI yearly, or sooner in case of suspected progression of the lesion on ultrasound. If imaging suggests HCC, we recommend referring patients for standard-of-care management.

Follow-up imaging after shunt closure

Since pre-closure imaging does not currently offer prognostic insight into nodule outcome after shunt closure, serial lifelong imaging is mandatory after shunt closure and should be performed every 3 to 6 months for 2 years, and yearly beyond that (Fig. 3). More frequent follow-up is probably warranted during puberty and pregnancy because of the potential impact of hormonal changes on tumour growth. Although closing a shunt may be associated with liver nodule regression or disappearance, there are anecdotal reports of patients presenting with HCC long after shunt closure. Therefore, until better

Key points

- Congenital portosystemic shunt(s) (CPSS) are often associated with systemic complications.
- A careful evaluation and lifelong surveillance of liver nodules is essential in patients with CPSS, before and after closure, as nodules of various histological types are highly prevalent in this population and may be malignant.
- Portopulmonary hypertension is the most life-threatening complication in patients with CPSS and requires regular monitoring both before and after shunt closure.
- Because of the major endocrine role of the liver, it is important to monitor potential hormonal abnormalities.
- As CPSS can have various clinical manifestations, a head-to-toe evaluation is essential at diagnosis.
- The anatomy of the shunt and the risk of portal hypertension after closure will determine the modality of closure.
- Predicting portal hypertension after shunt closure is difficult, but clinical factors may help in risk assessment.
- Closure is recommended in case of systemic complications or pre-emptively for extrahepatic CPSS.

risk stratification for the management of patients with CPSS and liver nodules is available, lifelong imaging is highly advisable. When there are doubts about features on imaging, a biopsy is imperative and the threshold for seeking a detailed histopathological examination must be low.

Recommendations – imaging

- Look for CPSS in patients with a liver nodule and no underlying liver disease.
- Initially characterise each nodule and perform longitudinal monitoring.
- Preferred imaging at baseline: hepatobiliary contrast-enhanced MRI/contrast-enhanced ultrasound.
- Pre-closure: perform imaging every 6 months (every 3 months if β-catenin activated or in doubt).
- Post-closure: perform imaging every 6 months or once a year for life (every 3 months in the first year if β-catenin activated and for longer if little or no regression, or if equivocal features).

Histology and molecular analysis

In patients with CPSS, nodule histology is uniquely characterised by its heterogeneity, and histological variability between patients is the norm. Further, a patient may present with numerous nodules, each differing in nature, such as FNH, HCA, and HCC. As described in Fig. 3, we recommend a biopsy for i) all liver lesions not meeting unequivocal radiological FNH criteria, ii) lesions increasing in size, and iii) lesions with evolving imaging characteristics.

In addition to the above, intratumoral heterogeneity is frequent in the setting of CPSS. Therefore, multiple biopsy samples of each nodule are recommended when feasible to minimise sampling bias. One report documented the synchronous presence of HCA, either β-catenin activated or hepatocyte nuclear factor 1α (*HNF1A*)-inactivated, and HCC in a single nodule.[6]

Given the unusual histological and molecular features of nodules in CPSS, current histopathological classification may not always be appropriate. For example, because the distinction between HCA and well-differentiated HCC may be impossible on a biopsy, a diagnosis of "well-differentiated hepatocellular neoplasm of uncertain malignant potential" may be favoured. Likewise, FNH lesions may not harbour all the characteristics seen in a liver with normal portal and arterial flow. Nonetheless, conventional histological terminology and descriptions do provide a framework to integrate clinical, imaging, and molecular findings.

In this complex context, molecular analysis and immunohistochemistry are strongly recommended since major discrepancies between histological subtyping and molecular data have been reported. β-catenin activation is frequently associated with a higher risk of malignant transformation, even in lesions with both classical histological and immunohistochemical FNH features. Available molecular data suggest a higher incidence of β-catenin-activated hepatocellular lesions in patients with CPSS than in the general population, where β-catenin-activated HCA represents 15% of all HCA cases.[20] Furthermore, in a single patient, multiple nodules displaying multiple *CTNNB1* (the gene encoding β-catenin) variants have been reported.[21] Therefore, assessing β-catenin activation is essential, using immunohistochemistry including the expression pattern of the surrogate marker glutamine synthetase.[22] More studies are needed to confirm whether the specific associations between immunohistochemistry pattern and risk of malignant transformation described in the general population also apply to CPSS-related tumours,[23] and whether they might predict nodule behaviour after shunt closure. For the time being, we recommend classifying CPSS-related tumours as "β-catenin activated" (Fig. 3) according to standard criteria,[22] irrespective of variants, for two reasons. First, the relative risk of different types of β-catenin activation in CPSS-related tumours is currently unclear, in particular regarding the potential for malignant progression of HCA with exon 7/8 *CTNNB1* variants, in addition to the known risk related to exon 3 variants.[24] Second, molecular testing may not always be available, or feasible, especially on small formalin-fixed and paraffin-embedded samples. Recommended minimum immunohistochemical stains and molecular workup are outlined in Table 2.

Recommendations – histology & molecular analysis

- Biopsy indications: i) nodules that do not meet classical FNH criteria, ii) nodules that increase in size or show evolving imaging features, iii) nodule heterogeneity, iv) nodules that show hypointensity on delayed hepatobiliary contrast-enhanced MRI.
- When performing biopsy of a nodule, also obtain biopsy of the non-nodular liver.
- Histology: i) follow current conventional diagnostic criteria, ii) make no diagnostic assumptions on other nodules from the same patient, iii) consider the possibility of underlying disease in the non-nodular liver.
- Immunohistochemistry: perform on each nodule whenever feasible or alternatively on selected nodules (minimum panel: β-catenin and glutamine synthetase).
- Molecular analysis: perform HCA subtyping and assess *TERT* promoter mutations whenever feasible.

[34]*Université Paris Cité, CRI, INSERM, Paris, France;* [35]*AP-HP, Département de Radiologie, Hôpital Beaujon. Nord, Clichy, France;* [36]*Pediatric Cardiology Unit, Department of pediatrics, Gynecology and Obstetrics, University of Geneva, Geneva, Switzerland;* [37]*Centre Universitaire Romand de Cardiologie et Chirurgie Cardiaque Pédiatrique, University of Geneva and Lausanne, Switzerland;* [38]*Department of Pediatric Endocrinology, Amsterdam University Medical Centers, University of Amsterdam, Amsterdam, The Netherlands;* [39]*AP-HP, Unité d'hépatologie pédiatrique et transplantation hépatique, Hôpital Necker, Paris, France;* [40]*Centre de Référence des maladies rares du foie de l'enfant, FILFOIE, France;* [41]*Swiss pediatric Liver Center, Division of pediatric surgery, Department of Pediatrics, Gynecology, and Obstetrics, University of Geneva, Geneva, Switzerland*
[†]*Contributed equally*

* Corresponding author. Address: Swiss Pediatric Liver Center, Gastroenterology, Hepatology and Pediatric Nutrition Unit, Department of Pediatrics, Gynecology and Obstetrics, University of Geneva, Geneva, Switzerland.
E-mail address: valerie.mclin@hcuge.ch (V.A. McLin).

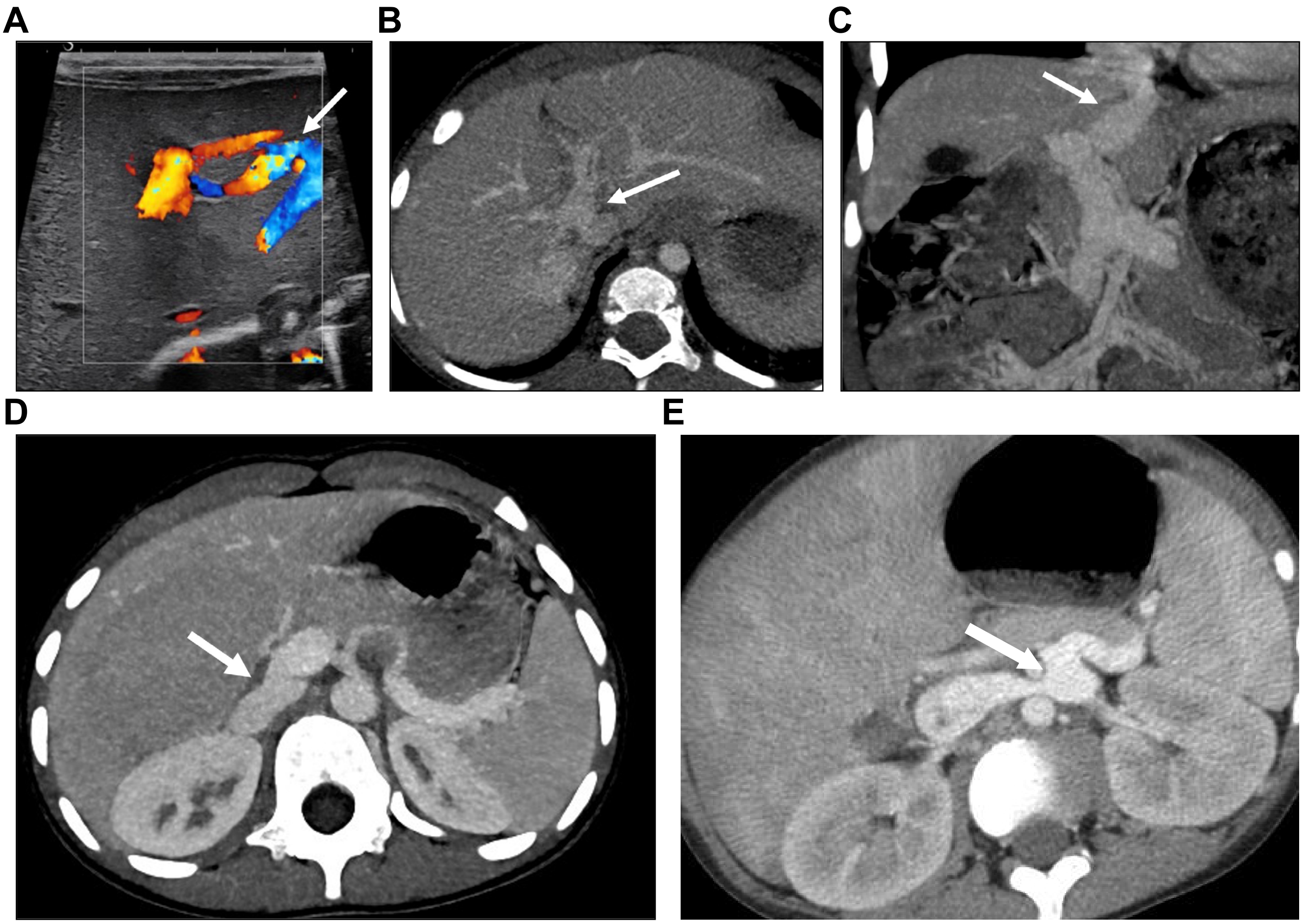

Fig. 1. Five anatomic forms of congenital portosystemic shunts. (A) Colour-Doppler ultrasound of the liver in a newborn showing a direct communication between the left portal vein and the left hepatic vein (arrow) consistent with an intrahepatic portosystemic shunt. (B) Axial image of contrast-enhanced computed tomography in the portal phase showing a wide side-to-side extrahepatic communication between the portal bifurcation and the inferior vena cava (arrow). (C) Coronal reconstruction of contrast-enhanced computed tomography in the portal phase showing an abnormal, large ductus venosus (arrow). (D) Axial image of contrast-enhanced computed tomography in the portal phase showing a wide end-to-side extrahepatic communication between the origin of the main portal vein and the inferior vena cava (arrow). (E) Axial image of contrast-enhanced computed tomography in the portal phase showing a wide side-to-side extrahepatic communication between the splenic vein and the left renal vein (arrow).

Table 1. Complications reported in CPSS.

Complication	Range	References
Pulmonary vascular complications		
PoPH	7 to 14% (67%*)	1–9
HPS	3 to 12%	1,4–9
Unspecified	2 to 28%	1,10
Liver nodule - any type of which % malignant (HB and HCC)	0 to 73% (of which 0 to 83% malignant and 6 to 63% premalignant)	1,3–6,8–13
Neurological complications	14% to 73%	1–6,8–12
Endocrine/metabolic/growth	14 to 67%	2,3,8,10
Haematology	9 to 33%	2,12,13
Cholestasis/Hyperbilirubinemia	9 to 73%	3,4,8,9,12,13
Other**	Sporadic	2,3,7,8,10,12,13

HB, hepatoblastoma; HCC, hepatocellular carcinoma; HPS, hepatopulmonary syndrome; PoPH portopulmonary hypertension.
This table combines reports of complications in extrahepatic and intrahepatic cases. The following references include patients aged 18 and over at diagnosis:[1,2,5,6,8,11] Percentages (%) express fraction of patients in a given series.
* Overestimated prevalence due to selection bias.
** Pancreatitis, microangiopathic haemolytic anaemia, glomerulonephritis, vaginal bleeding, protein losing gastropathy, coagulopathy, gastrointestinal bleeding, intrauterine growth retardation, abdominal symptoms, isolated neonatal respiratory distress.

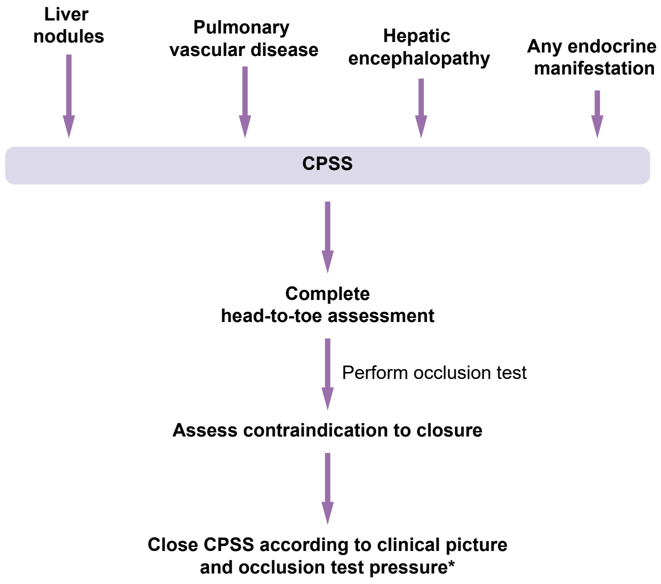

Fig. 2. Approach to the patient with suspected CPSS. *If portopulmonary hypertensionn present, treat according to recommendations prior to CPSS closure. CPSS, congenital portal systemic shunt(s).

Management of patients with liver nodules and CPSS

Given the unpredictable biological and histological features of nodules in the subset of patients with CPSS, it is recommended to close any CPSS associated with a liver mass, independently of patient age, bearing in mind that tumour behaviour following closure is equally unpredictable.[25] Closing the shunt may allow for regression and/or disappearance of the nodule with time, by restoring normal portal and arterial flows, thereby rendering surgery unnecessary. Should the nodule require resection after shunt closure, multiphasic contrast-enhanced CT is essential to reassess vasculature prior to surgery. Exceptionally, and on a case-by-case basis, nodule resection may be performed concurrently with shunt closure.

Evaluating the indication and timing of nodule excision is complex, and must be based on histological, molecular, and anatomical findings. The decision should be multidisciplinary and dependent on resources and expertise. When doubt persists regarding sampling or risk of malignant degeneration of a nodule, surgical tumour resection should be considered without delay. Malignant CPSS-associated liver masses require standard oncological management in addition to shunt closure[26] (Fig. 3).

Approach to the patient with portopulmonary hypertension and a CPSS

CPSS are associated with cardiopulmonary complications (Table 1), which have been reviewed elsewhere.[25] The most life-threatening complication is portopulmonary hypertension (PoPH), a subtype of pulmonary arterial hypertension (PAH) resulting from liver disease or portosystemic bypass, the pathophysiology of which is not fully understood. The management of these patients is notoriously complex. In this section we will use PoPH to refer to pulmonary arterial hypertension in the setting of liver disease or portosystemic bypass, PAH for what is known in the field of pulmonary arterial hypertension in general, and pulmonary hypertension in the few instances where both pulmonary arterial and post-capillary hypertension are considered.

Diagnosis and screening

The prevalence of PoPH in patients with CPSS ranges from 7% to 14% (Table 1). As epidemiological data are scarce, the prevalence and recommendations reported here are based on retrospective observational studies performed by expert centres. Systematic screening of all patients is recommended at the time of diagnosis of CPSS, and yearly thereafter until 1 year after shunt closure if PoPH was absent pre-closure. However, while the threat of PoPH probably ceases after CPSS closure, annual PoPH follow-up after closure is still recommended in the presence of liver disease or portal hypertension.

Transthoracic echocardiography (TTE) is the first-line, non-invasive screening tool of choice. TTE also allows for the detection of CHD, another risk factor for PAH which has been reported in 17% of individuals in a cohort of 168 patients with CPSS.[7] In addition, patients with hypoxemia or increased alveolo-arterial oxygen gradient may benefit from contrast-enhanced echocardiography to detect intrapulmonary vascular dilatations indicative of hepatopulmonary syndrome, which may – rarely – develop in combination with PoPH.

Patients with echocardiographic criteria characteristic of an intermediate or high probability of PAH according to the European guidelines should be referred to expert centres for right heart catheterisation to confirm diagnosis and help guide treatment.[27] The most recent definition of PAH is a measured mean pulmonary arterial pressure >20 mmHg, pulmonary capillary wedge pressure ≤15 mmHg and calculated pulmonary vascular resistance (PVR) >2 Wood units (WU), or 3 WU.m^2 in children.[27] A diagnostic algorithm is detailed in Fig. 4.

Recommendations - screening

- Perform TTE at time of CPSS diagnosis in all patients to screen for PoPH.
- Perform TTE at least once in the first year after shunt closure, and yearly thereafter if portal hypertension develops.
- To confirm PoPH diagnosis and assess its severity, refer patients with an intermediate or high probability of pulmonary hypertension on TTE for right heart catheterisation.
- Rule out congenital heart disease at initial TTE in all patients with CPSS.
- For patients with hypoxemia, perform contrast-enhanced echocardiography to detect intrapulmonary vascular dilatations indicative of hepatopulmonary syndrome.

Management

Optimal management of patients with PoPH requires a multidisciplinary approach involving experts in both PAH and liver disease to determine i) timing of shunt closure, ii) medical PAH therapy or iii) the indication for lung, liver, or combined transplantation in the most severe cases (summarised in Fig. 5).

Medical management of PoPH in the setting of CPSS does not differ significantly from other causes of PAH. Endothelin receptor

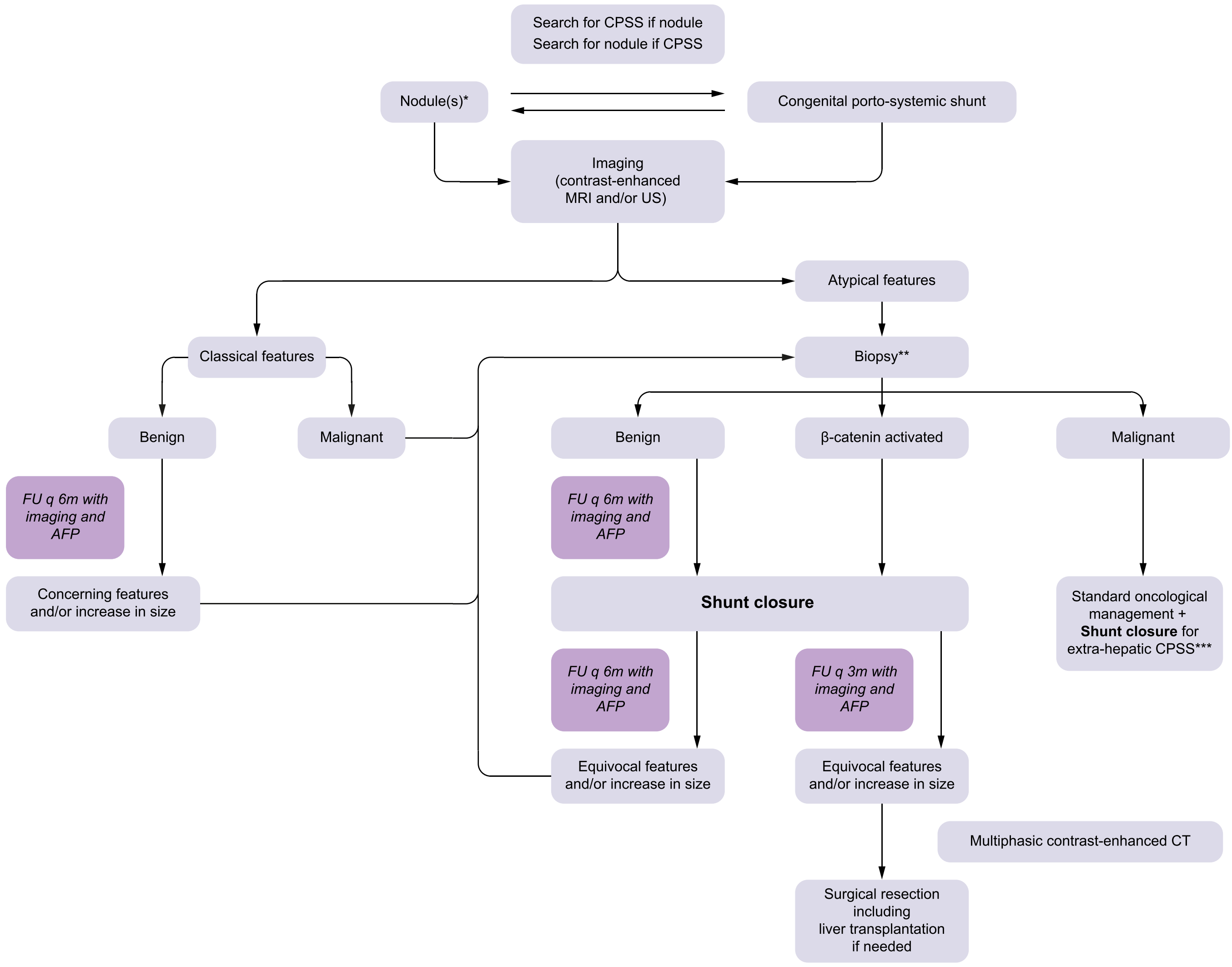

Fig. 3. Management algorithm in the presence of a liver mass(es) and/or CPSS. *Management algorithem applicable to each liver module. **Beware of intra-nodular heterogeneity or sampling error. ***See section on contraindications to surgical or endovascular closure. AFP, alpha-fetoprotein; CPSS, congenital portal systemic shunts; FU, follow-up; US, ultrasound.

antagonists, phosphodiesterase-type 5 inhibitors, soluble guanylate cyclase stimulators, and prostacyclin analogues aim to reduce PVR, improve right ventricular function, and ultimately facilitate normal activities and improve survival, as has been reported in other causes of PAH.[28–31]

Shunt closure is recommended for all patients with CPSS and PoPH. If surgical closure is planned, anaesthetic risk must be assessed on a case-by-case basis according to PoPH severity and the presence of right ventricular failure. Initiating PAH-specific medical therapy can be useful before closure to mitigate the risk of peri-interventional worsening of PoPH. However, it should be emphasised that the impact of CPSS closure on the evolution of PAH varies from one patient to another. Closing the shunt does not guarantee the reversal of pulmonary vascular remodelling. Conversely, however, it is now accepted that PoPH will not regress if the trigger remains. Various factors, such as the duration of pulmonary vascular involvement, may impact the outcome, as most cases of reversibility have been reported in young children.[3,5,7,32] In addition, PAH persistence or progression will also be driven by a potentially uncorrected CHD. For the small subset of patients with CPSS and associated CHD and normal or near-normal PVR, intracardiac shunt closure may be considered to reduce systemic-to-pulmonary shunting. Operability criteria have been proposed by paediatric and adult experts, but robust data on haemodynamic predictors are lacking.[33,34]

In some cases, transplantation may be considered to treat patients with CPSS and PoPH. Lung and/or liver transplantation (LT) may be discussed on a case-by-case basis if PoPH and/or liver disease are still potentially life-threatening despite shunt closure and maximal PAH therapy, or before shunt closure in the presence of advanced liver disease (cirrhosis). Double lung transplantation can be considered as in end-stage idiopathic PAH, despite maximal medical therapy, in highly selected patients, provided there is no persistent liver disease or portal hypertension. While double lung transplantation is now the method of choice for most patients with PAH, heart-lung transplantation is only considered for patients with associated complex CHD.[35]

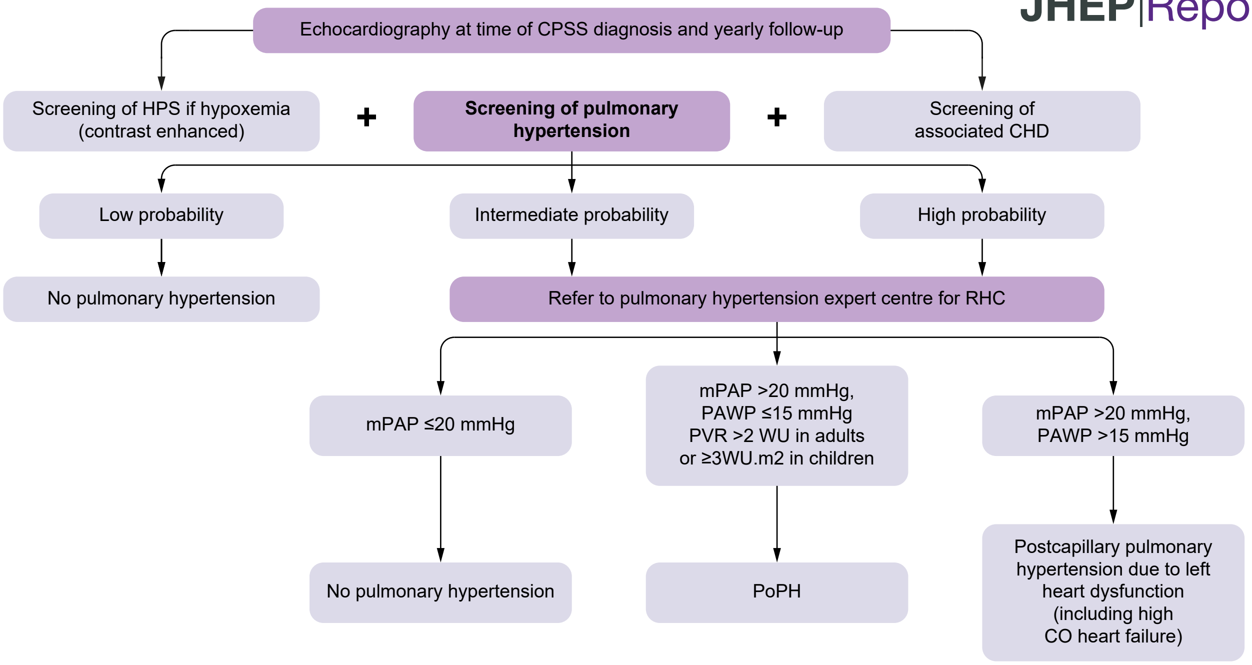

Fig. 4. Diagnostic algorithm for all forms of pulmonary hypertension in patients with CPSS. Echocardiographic probability of pulmonary hypertension is based on the value of the tricuspid regurgitation velocity and the detection of other echocardiographic signs suggestive of pulmonary hypertension. Low probability of pulmonary hypertension: tricuspid regurgitation velocity ≤2.8 m/s and no other echo pulmonary hypertension signs. Intermediate probability of pulmonary hypertension: tricuspid regurgitation velocity ≤2.8 m/s with echo pulmonary hypertension signs, or tricuspid regurgitation velocity 2.9-3.4 without other echo pulmonary hypertension signs. High probability: all other conditions. CHD, congenital heart disease; CO, cardiac output; CPSS, congenital portal systemic shunts; HPS, hepatopulmonary syndrome; mPAP, mean pulmonary arterial pressure; PAWP, pulmonary arterial wedge pressure; PoPH, portopulmonary hypertension; PVR, pulmonary vascular resistance; RHC, right heart catheterisation; WU, Wood units.

In contrast, for patients with PoPH who have pre-existing liver disease or develop liver disease (*e.g.* portal cavernoma following shunt closure) or portal hypertension, LT may be considered, but only if PoPH is not severe and right ventricular function is preserved. The use of PAH-specific medical therapies as a bridge to LT in adults or children has proven effective to reach haemodynamic criteria, allowing for safe transplantation in most cases (mean pulmonary arterial pressure <35 mmHg with PVR <5 WU or mean pulmonary arterial pressure >35 mmHg with PVR <3 WU).[36] LT may have a beneficial

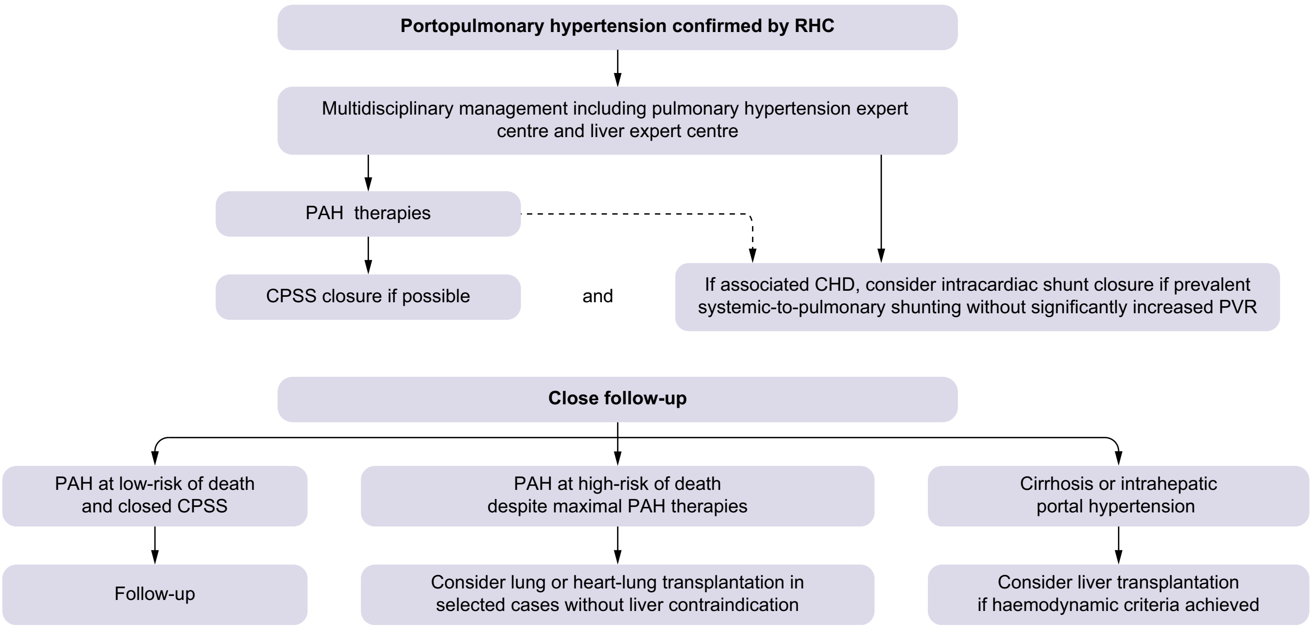

Fig. 5. Treatment algorithm of portopulmonary hypertension associated with CPSS. CHD, congenital heart disease; CPSS, congenital portal systemic shunts; PAH, pulmonary arterial hypertension; PVR, pulmonary vascular resistance; RHC, right heart catheterisation.

Table 2. Recommended histological, immunohistochemical and molecular workup of liver nodule(s) in patients with congenital portosystemic shunts.

	Recommendations
Recommended routine stains	Haematoxylin/eosin<br>Reticulin stain
Recommended immunohistochemistry	Glutamine synthetase<br>β-catenin<br>SAA (serum amyloid A)<br>CRP (C-reactive protein)<br>CD34<br>Glypican-3LFABP<br>(liver-fatty acid binding protein)
Recommended minimal molecular work-up	β-catenin activation (*CTNNB1* exon3, 7/8)<br>*TERT* promoter mutation
Recommended sample size	Needle gauge should be sufficient to provide adequate diagnostic yield according to local laboratory standards.

effect on long-term survival in cirrhotic adults with PoPH and in extrahepatic CPSS.[10,30,37–39] There are no reports of combined liver-lung transplantation for life-threatening pulmonary vascular disease and liver disease.

Recommendations - management

- Use a multidisciplinary approach involving expertise in both PAH and CPSS to manage patients with PoPH.
- For all patients with PoPH, close CPSS to remove the insult to the pulmonary vasculature.
- Manage PoPH due to CPSS like other forms of PAH.
- For highly selected patients with a closed CPSS and no liver disease or portal hypertension, consider double lung transplantation in end-stage PoPH despite maximal therapy.
- In patients with PoPH who develop liver disease and/or portal hypertension, consider LT as a therapeutic option, provided requisite haemodynamic criteria are met.

Endocrine manifestations

As the liver plays a major endocrine role, patients with CPSS may show aberrations in hormone metabolism owing to portosystemic bypass, albeit partial. Not only does the liver produce several hormones, but it is also responsible for the first-pass metabolism of insulin, sex hormones, glucocorticoids and mineralocorticoids. In addition, the liver produces substrates that are required for hormone synthesis, such as cholesterol and lipoproteins for glucocorticoid synthesis. Although somewhat beyond the scope of a position paper, the purpose of this section is to give a succinct overview of the liver's role in the main endocrine pathways, including evidence from animal models or other situations of portosystemic bypass, to increase awareness and support our recommendations (summarised in Table 3).

Thyroid function

Hypothyroidism, characterised by low free T4 and normal thyroid stimulating hormone –(mimicking central hypothyroidism) has been reported in CPSS.[40] Putative pathophysiological mechanisms include decreased first-pass metabolism of T4 and reduced hepatic thyroxine-binding globulin synthesis.[17,41] In addition, thyroxine-binding globulin synthesis is partially dependent on oestrogen, which itself is metabolised in the liver.[42] We recommend performing diagnostic tests for hypothyroidism in every patient during the pre-closure workup.

Glucose homeostasis

Hyperinsulinaemic hypoglycaemia is one of the cardinal signs of CPSS and should prompt the workup for CPSS. Hypoglycaemia is particularly problematic in the neonatal period, as the developing brain is particularly vulnerable owing to a lack of alternative fuel in the form of ketones.[3,17,40,43] Symptoms can be difficult to distinguish from minimal hepatic encephalopathy. In pre-school children, symptoms of hypoglycaemia can be non-specific (behavioural problems, moodiness, exercise intolerance and fatigue). We recommend diagnostic tests for hyperinsulinaemic hypoglycaemia in every pre-school-aged patient or in patients for whom there is a clinical suspicion during the pre-closure work-up.

Linear growth

CPSS seems to impact linear growth in two different ways. While short stature and intrauterine growth retardation[44–48] have been reported in CPSS, anecdotal evidence also points to tall stature in childhood.[17] The pathophysiology underlying these effects on linear growth is unclear, but surgical portosystemic shunting has long been known to improve linear growth in children with glycogen storage disease.[49] Putative mechanisms include dysregulation of insulin-like growth factor 1 and growth hormone signalling or the impact of hyperandrogenism (see below).

Adrenal function

There is mounting evidence of hyperandrogenism in CPSS. Stigmata of hyperandrogenism include premature adrenarche, hirsutism, virilisation, menstrual irregularities, and subfertility.[17,50] In addition, hyperandrogenism leads to accelerated somatic maturation, accentuated linear growth, and precocious puberty.[17,40] In patients with CPSS, hyperandrogenism is thought to occur because of incomplete or partial hepatic sulfation of DHEA (dehydroepiandrosterone) to the less active DHEA-S (dehydroepiandrosterone sulphate), thereby leading to a higher proportion of potent circulating androgens.[42,51] Testing for hyperandrogenism is recommended in both adults and children with CPSS. Conversely, CPSS may be considered in cases of unexplained hyperandrogenism, especially with a relatively high fraction of DHEA compared to DHEA-S. There is no data on how CPSS may impact fertility, although hyperandrogenism may be involved.

Relative adrenal insufficiency (RAI) can occur in children with decompensated cirrhosis.[52] RAI may be caused by lipoprotein substrate deficiency (adrenal exhaustion syndrome), multifactorial hypothalamus-pituitary-adrenal axis impairment (cytokine storm, bacterial translocation, endotoxemia), decreased hepatic synthesis of cortisol binding globulin, adrenal haemorrhage in coagulopathy and adrenal steal phenomenon in portal

Table 3. Recommended pre-closure endocrine assessment in patients with CPSS.

System	Laboratory and clinical assessment
Thyroid[1]	TSH, Free T4, total T4, TBG, free T3, total T3, reverse T3, thyroglobulin
Glucose homeostasis[1]	**In pre-school patients or patients with clinical suspicion of hypoglycaemia:** fasting glucose and glucose measurements 1.5 – 2 – 2.5 h after a carbohydrate rich meal (when available preferably continuous glucose monitoring for several days). **In case of hypoglycaemia (<2.5-3.0 mmol/L):** insulin, ketones and free fatty acids at time of hypoglycaemia.
Adrenal[1]	**In case of clinical suspicion of hyperandrogenism***:* bone age, IGF-1, IGF-BP3, LH, FSH, testosterone, estradiol, DHEA, DHEA-S, androstenedione. **In case of clinical suspicion of adrenal insufficiency:** morning cortisol followed by synacten stimulation. Diagnosis according to standard criteria (morning cortisol and/or synacten test).
Growth[2]	Measure and plot weight and height + parental height. Tanner staging

CPSS, congenital portosystemic shunts; DHEA, dehydroepiandrosterone; DHEA-S, dehydroepiandrosterone sulphate; FSH, follicle stimulating hormone; IGF, insulin growth factor; LH, luteinizing hormone; TBG, thyroid binding globulin; TSH, thyroid stimulating hormone.
[1] Repeat post-closure in case of abnormal findings.
[2] Repeat post-closure irrespective of findings post-closure.

hypertension.[53] However, the clinical effects of an excess or a deficiency of cortisol have not yet been reported in patients with CPSS. Adrenal insufficiency can also cause hypoglycaemia, one of the clinical signs associated with CPSS (see above). Symptoms are non-specific and can be difficult to distinguish from minimal hepatic encephalopathy. We recommend performing diagnostic tests for adrenal insufficiency in the pre-closure work-up as it is difficult to tease out the relative contribution of hyperinsulinism, RAI, and hepatic encephalopathy.

Planning shunt closure: pre-operative workup

Careful pre-operative assessment is essential for several reasons. First, understanding exact shunt anatomy, pressures, and flow direction in complex cases ensures a personalised and safe approach to safeguard outcomes. This means careful management of extrahepatic complications prior to shunt closure, and assessing how these modify the shunt 'phenotype' between diagnosis and closure. In addition, an accurate pre-operative workup helps mitigate procedural risks, and possibly, the risk of portal hypertension, while offering patients and their relatives anticipatory guidance. A thorough head-to-toe approach to patients with CPSS is summarised in Table 4.

Pre-operative imaging

Once the multiple complications of CPSS have been identified, consideration can be given to the timing of shunt closure and which technical approach to use. As previously stated, any of the medical complications summarised herein are an indication for shunt closure.

The first point is to understand shunt anatomy, which is extremely variable (Fig. 1). It is best assessed using abdominal Doppler ultrasound, angio-CT, contrast-enhanced MRI, or a combination of these techniques. Sometimes, the shunt may be located in close proximity to important surrounding vessels, such as the splenic vein, renal vein and inferior vena cava. Therefore, detailed phlebography of the inferior vena cava, splenic and renal veins is essential to map shunt anatomy and plan closure.

Next, it is necessary to determine the treatment strategy. During the phlebography, an occlusion test of the CPSS is essential to determine the feasibility of closure, the optimal closure method, and the required number of closure stages. Such investigations enable assessment of the anatomy and size of both the main portal vein and its intrahepatic branches, and of whether the shunt is simple or complex. A simple shunt has one portosystemic communication, while a complex shunt has more

Table 4. Head-to-toe evaluation at CPSS diagnosis/pre-closure and post-closure follow-up.

System	Basic workup*	Post-closure follow-up
Cardio-pulmonary	Transthoracic echocardiography<br>• Screen for PoPH and assess right ventricular function at time of CPSS diagnosis and yearly thereafter. Right heart catheterization if intermediate or high probability of PoPH on echocardiography.<br>• Look for associated congenital heart disease.<br>• Use contrast-enhanced echocardiography to look for HPS if suspected.	In case of PoPH, long-term follow-up at least every 6 months.<br>If no PoPH, screening until 1 year after CPSS closure.<br>In case of HPS, periodic assessment by pulse oximetry, and bubble echocardiography to assess resolution once pulse oximetry is normal, or if resolution is slower than expected.
Liver	Doppler ultrasound, LFT, sBA, CT angiogram, portal angiography and occlusion test, nodule biopsy (*),<br>non-nodular liver biopsy. In newborn, add galactosemia screen.	Follow nodules post closure depending on histopathological subtype – lifelong-evaluation of portal vessels (expansion, portal hypertension).
Renal†	Urinalysis (for haematuria, proteinuria).	Repeat post closure.
Endocrine	Clinical exam, explore all axes (history and clinical signs)*.	Repeat abnormal axes.<br>Follow growth.
GI/GU	Thorough history with workup based on symptoms.	Clinical follow-up.
CNS†	Neurocognitive evaluation, T1 weighted MRI (globus pallidus), plasma NH_3.	Re-assess after 1-2 years.

CNS, central nervous system; CPSS, congenital portosystemic shunts; GI, gastrointestinal; GU, genitourinary; HPS, hepatopulmonary syndrome; LFT, liver function test; PoPH, portopulmonary hypertension; sBA, serum bile acid quantification.
* Details in text.
† Not discussed in current text, added for practical reasons [4,17, reviewed in 26], focus of another symposium/recommendations.

than one. In addition, an occlusion test is central to measuring portal pressure upon temporary closure of the shunt. In most patients whose main portal vein or intrahepatic portal veins are not visible on non-invasive imaging, this approach allows for the visualisation of a hypoplastic portal vein.

Biopsy of the non-nodular liver

The fact that patients with CPSS show abnormal non-nodular liver histology is now widely accepted. Key findings include the presence of dilated thin-walled portal vein branches or a combination of lymphatics and portal vein branches, the absence of small portal venules, portal arterial-biliary dyads, and increased arterial profiles in the portal tracts, in line with the known compensatory mechanism of blood flow. Other histological findings may be associated with the type of CPSS and the degree of arterial buffer response.[54]

Consequently, in addition to comprehensive nodule sampling if present (see above), we recommend a biopsy of the non-nodular liver according to current standards.[55] The rationale is threefold. First, to evaluate vascular alterations, fibrosis and architectural changes which may impact closure strategy and outcome.[54,56,57] Second, to rule out other causes of liver disease and to inform management decisions. Specifically, in CHD cases, a liver biopsy can help determine whether to close the shunt, given the additional post-hepatic vascular insult. Third, provided there is appropriate ethical approval and consent, it is of interest for research purposes, and will help us to understand the potential prognostic value of these samples.

Recommendations

- Determine shunt anatomy and intrahepatic/extrahepatic portal anatomy with imaging.
- Perform an occlusion test to determine the feasibility of closure, the optimal closure method and the required number of closure stages.
- Perform biopsy of the non-nodular liver: i) to evaluate vascular alterations and architectural changes, ii) to rule out other causes of liver disease (*i.e.* CHD), and iii) for research purposes.

Shunt closure: approach and timing

Approach

Upon preparing for shunt closure, two important decisions must be made. First, choosing between the endovascular and surgical approach. In broad terms, long shunts can generally be closed by endovascular methods, as available devices will safely hold in place. Shorter shunts may be more easily and safely closed surgically. The width of the shunt is less critical, as some cardiac devices can be securely deployed in shunts with a wide diameter. We recommend closing the shunt using the least invasive approach, with a careful risk/benefit evaluation, considering individual anatomy (simple or complex), local expertise, device availability (Table 5), and risk estimation of secondary portal hypertension.

The second decision is determining the safest approach between a one-stage or a two-stage closure (Table 5). Briefly, the concept behind a two-stage closure is protecting the patient from acute, severe portal hypertension. The basic principles of a surgical approach are to a) measure porto-mesenteric pressure in a jejunal vein following temporary shunt closure intraoperatively, and b) observe the bowel for colour change due to venous stasis – emphasising that this is only possible during surgical closure. During the preoperative occlusion test, it is recommended to measure the portosystemic pressure gradient, which is more clinically significant and reliable than absolute portal pressure (see supplementary material). An absolute mesenteric pressure >30 mmHg or a portosystemic gradient >20 mmHg on an occlusion test is considered by some centres to be an absolute indication for a two-stage shunt closure, to minimise the consequences of acute portal hypertension. Either a one-stage or two-stage closure can be considered for portosystemic gradients between 10 and 20 mmHg, depending on anatomic and clinical features. The definitive decision to close in one stage or two stages should be taken during laparotomy or angiography.

For endovascular approaches, commercially available endovascular devices include vascular plugs specifically labelled to occlude vessels of variable size, and cardiovascular devices designed to occlude ductal, atrial, and ventricular septal defects. The latter are used off-label for CPSS closure. As no specific device has been developed to allow for a two-stage endovascular closure, endovascular options are tailor-made. Tailor-made devices include reduced stents, perforated ventricular septal occluders with stent grafts and cut microvascular plugs with coils. Tailor-made endovascular approaches for two-step closure may be feasible, but this approach needs to be discussed on a case-by-case basis and considered in expert centres when the benefits outweigh the risks.[58–62]

For the surgical approach, the essential steps are *in situ* measurement of the portosystemic gradient and assessing the bowel's response to the invasive occlusion test. Indeed, during the first step of a planned two-stage closure, findings may differ from the results of the occlusion test on angiography, thus allowing for a shift towards a one-stage closure. This change in assessment and plan is less likely to occur with two-stage endovascular closure which relies only on pressure measurement. On the other hand, it is advisable to opt for a two-stage approach by performing a partial surgical ligation process if the portosystemic gradient is >20 mmHg, if the absolute portal pressure is >30 mmHg, or if the bowel appears dusky. Following partial shunt ligation, the patient is followed at regular intervals by Doppler ultrasound, and complete shunt closure is considered, either by re-laparotomy or using an endovascular approach, 3 to 6 months after the first laparotomy, provided complete occlusion has not occurred spontaneously in the interim [described in ref [63]].

Several points warrant careful attention after shunt closure. For wide shunts, a risk of device migration has been reported,[64,65] with an increased likelihood of complications with tailor-made devices. This is monitored using daily Doppler ultrasound in the first few days after closure. Further, it is important to emphasise that regardless of the method, the risk of thrombosis upstream of the occlusion is of concern, and can be easily assessed by daily Doppler ultrasound. Therefore, prophylactic anticoagulation according to local protocols must be initiated at the time of closure. Management of anticoagulation should be adapted in case of a coagulopathy, or if there is evidence of thrombus extension. Importantly, secondary intrahepatic portosystemic shunts may open after primary shunt closure, be it endovascular or surgical. These shunts are either novel shunts not seen on the occlusion test, or part of complex shunts comprising more than one portosystemic

Table 5. Suggested approach to closure based on shunt type.

	One-stage	Two-stage
Intrahepatic CPSS	Endovascular (vascular plugs and coils)	Uncommon
Patent ductus venosus	Endovascular (vascular plugs; septal, duct and muscular occluders)	Uncommon
Extrahepatic side-to-side and end-to-side (from portal vein to inferior vena cava) CPSS	Endovascular (vascular plugs; septal, duct and muscular occluders, covered stents)<br>Consider surgery if complex anatomy or wide and short shunt.	Surgical<br>Consider endovascular with tailor-made devices if complex surgery or for the second procedure after surgical banding.
Extrahepatic CPSS upstream of portal vein	Endovascular (vascular plugs and coils, covered stents)<br>In short shunts, consider surgery, especially in younger patients.	Surgical

CPSS, congenital portosystemic shunt(s).

communication. They may act as 'pop-off' valves protecting the patient from acute portal hypertension. Longitudinal follow-up is essential to observe whether these secondary shunts close spontaneously, or are of clinical relevance, in time requiring their own closure.

Rarely and in exceptional cases, LT may be considered on a case-by-case basis as a method of closure for highly complex and exceptional situations including refractory recurrence of intrahepatic shunts, or nodules too numerous to count and/or unfavourable histology in one or several nodules of a liver with multiple nodules (see section below on contraindications to closure).

Timing of CPSS closure

There is no formal consensus on the timing of closure. A CPSS can be closed at any age depending on the clinical presentations described below.

For intrahepatic CPSS diagnosed at birth or *in utero*, it is generally recommended to wait for spontaneous closure during the first 2 years of life, provided there are no significant clinical complications.[43,66,67] If the shunt does not close spontaneously and is still patent in the second year of life, or if the patient experiences systemic complications of portosystemic shunting regardless of age, the consensus is to close the shunt.

For extrahepatic CPSS, pre-emptive closure even in asymptomatic patients is the consensus, as they are unlikely to close spontaneously and are associated with more severe complications.[66] This will likely protect from potentially irreversible clinical complications, such as those discussed above.[6] Additionally, there is a growing body of evidence that CPSS are associated with severe neurocognitive and psychiatric complications[9,68] which are accepted to be the equivalent of chronic portosystemic encephalopathy. For now, clinical experience suggests that CPSS exposes patients to neurocognitive impairment and therefore should be closed as soon as reasonable, including in neonatal management. However, this exceptional indication is limited to the rare cases in which compromised hepatopetal flow may be associated with portal involution, and to centres with the requisite multidisciplinary expertise.

The diagnosis of CPSS is increasingly being made prenatally.[69,70] Possible foetal CPSS complications include intrauterine growth retardation, cardiomegaly, or even cardiac failure and hydrops fetalis, justifying prenatal follow-up.[71] In case of severe complications, optimal timing for delivery will be managed by the obstetrician, and delivery in a centre with a neonatal intensive care unit is recommended. Recent observations suggest that even in extrahepatic CPSS, umbilical vein blood maintains an intrahepatic portal venous system in the foetus, although it might be difficult to observe because of the vascular steal phenomenon. After birth and the end of umbilical circulation, this network may regress with the risk of complete portal atrophy or thrombosis in severe cases.[69] Therefore, in the absence of intrahepatic portal flow after birth in an infant with a prenatal diagnosis of extrahepatic CPSS, recent reports suggest that neonatal closure may be the best strategy to redirect flow to the portal venous system to prevent its involution (unpublished communication from Prof. S. Franchi-Abella). This novel approach requires prenatal diagnosis, careful anticipation, and collaboration between obstetrics, neonatology and interventional radiology in a highly specialized center, to offer the infant immediate neonatal assessment and closure.

Recommendations

- Follow all asymptomatic intrahepatic CPSS detected at birth longitudinally until spontaneous closure and 1 year beyond documented closure.
- Close asymptomatic intrahepatic CPSS if they do not close spontaneously within the first 2 years of life.
- Close all asymptomatic extrahepatic CPSS pre-emptively, as early as possible depending on local resources.
- Close all symptomatic CPSS beyond the neonatal period.
- In case of prenatally detected CPSS, perform prenatal and neonatal assessment in a specialised centre, since closure of an extrahepatic CPSS may be indicated early in the postnatal period.

Predictors of portal hypertension following closure

It is difficult to predict who will develop portal hypertension after shunt closure, as data are lacking. Although none has been validated, potential predictive factors include absent portal venules on non-nodular liver histology, syndromic forms of CPSS associated with liver diseases (*i.e.* porto-sinusoidal vascular disease, cardiac fibrosis/cirrhosis), absence of visualisation of the

Table 6. Risk stratification for predicting post-closure symptomatic portal hypertension.

Risk level	CPSS	Visibility of portal vein	Cardiac disease
Very low	Isolated intrahepatic CPSS or patent ductus venosus	Visible main portal vein and/or intrahepatic portal veins	No cardiac disease
Low	Isolated CPSS	Main portal vein visible only on occlusion test	No cardiac disease
Intermediate	Syndromic CPSS	Main portal vein visible or not only on occlusion test	No cardiac disease
High	CPSS	Main portal vein visible or not only on occlusion test	Congenital or acquired heart disease with high cardiac pressure

CPSS, congenital portosystemic shunts.

portal system on an occlusion test, a portal pressure >30 mmHg or a portosystemic gradient >20 mmHg on an occlusion test. In Table 6, we suggest an approach for predicting the risk of portal hypertension following CPSS closure, based on unpublished single-centre experience.

Contraindications to surgical or endovascular closure

There are two formal contraindications to closing a CPSS. First, a CPSS should not be closed if a severe underlying liver disease is present and will most probably not be improved by shunt closure. In such a case, we recommend LT as the treatment of choice, rather than the prolonged management recommended above in the absence of associated significant liver disease. Second, if a large and/or multifocal malignant liver tumour(s) such as HB or HCC is/are present, the patient might be best served by LT. The rationale is to avoid the morbidity associated with complex liver resection and the risk of compromised liver regeneration in the absence of normal portal venous flow. In addition, it can be argued that avoiding time-consuming serial treatment with shunt closure, chemotherapy and hepatectomy is in the interest of the patient by minimising complications and morbidity. Third and exceptionally, extremely complex shunt anatomy may preclude endovascular or surgical closure.

Relative contraindications to shunt closure include the presence of multiple pre-malignant lesions such as β-catenin-activated adenomas or lesions of questionable malignant potential. If the tumour can be treated by reduction therapy, and in addition if an occlusion test demonstrates a satisfactory portal vein, shunt closure may be performed with the expectation that the tumour will respond to both. However, a large tumour, or a multifocal malignancy – combined with a very small pre-occlusion portal vein that is not expected to expand after shunt occlusion – may be considered for LT in addition to standard-of-care oncological treatment.

Recommendations

- Consider LT rather than isolated CPSS closure in case of pre-existing liver disease which will continue to evolve despite shunt closure, if the child has liver disease severe enough to not tolerate shunt closure, or in rare cases of extremely complex anatomy not amenable to endovascular or surgical closure.
- Consider LT rather than isolated CPSS closure in case of unresectable lesions of questionable malignant potential or multifocal malignant liver tumour(s) and CPSS.
- Close the shunt if the tumour can be treated by reduction therapy and an occlusion test demonstrates a satisfactory portal vein.
- Consider LT and oncological treatment in case of a large tumour, or a multifocal malignancy, combined with a very small pre-occlusion portal vein that is not expected to grow after shunt occlusion.

Summary and conclusion

In conclusion, CPSS are associated with severe complications which can be challenging to manage. The crux of management is a thorough head-to-toe assessment for complications at the time of diagnosis, a multidisciplinary approach and prompt treatment of PoPH if present. Understanding shunt anatomy and portal vasculature, quantifying portal pressure and portosystemic gradient with an occlusion test, and evaluating nodule histology and size will inform the closure approach. Much is still unknown about the multisystem complications associated with CPSS. Until risk stratification is possible, longitudinal follow-up is required for all patients prior to shunt closure, as well as post closure for any patient with a liver nodule or PoPH.

Abbreviations

CHD, congenital heart disease; CPSS, congenital portal systemic shunt(s); FNH, focal nodular hyperplasia; HCC, hepatocellular carcinoma; LT, liver transplantation; NRH, nodular regenerative hyperplasia; PAH, pulmonary arterial hypertension; PoPH, portopulmonary hypertension; PVR, pulmonary vascular resistance; RAI, relative adrenal insufficiency; TTE, transthoracic echocardiography; WU, Wood units.

Financial support

The Meeting of Experts was funded by EJP RD NSS, SASL, ESPGHAN. The registry is sponsored by a registry grant from EASL and a network grant from ESPGHAN.

Conflict of interest

The authors declare no conflicts of interest that pertain to this work.

Please refer to the accompanying ICMJE disclosure forms for further details.

Authors' contributions

Conceptualization: McLin, Plessier, Wildhaber, Debray, Brütsch, Savale, van der Doef. Methodology: McLin, Wildhaber, Debray, Brütsch. Writing original draft: Wildhaber, Rougemont, Savale, Wacker, Lador, Debray, Brütsch, McLin, Sen Sarma, van Albada, Zwaveling, Quaglia, van der Doef. Reviewing: all authors. Funding acquisition: McLin.

Acknowledgements

The authors are grateful to the following bodies for their support of the symposium: the European Joint Program for Rare Disease Networking Support Scheme (EJP RD NSS), the Swiss Association for the Study of the Liver (SASL), and the European Society for Pediatric Gastroenterology and Nutrition (ESPGHAN), ERN RARE LIVER and the VALDIG group. Thanks also go out to the University of Geneva, the University Hospitals Geneva Communications Department, and Drs Barooty, Salamoni and Steger for logistical support. Finally, the authors are indebted to Cristina Späni and Jérôme Divorne for their invaluable contributions to the meeting of experts and to Simona Korff for her management of the IRCPSS. The Registry is supported by ESPGHAN and the European Association for the Study of the Liver (EASL).

In addition, the authors are indebted to the following participants for their valuable clinical contributions and discussions: Ana Baiges, Hepatology, Hospital Clínic de Barcelona, Barcelona, Spain; Ron Bardin, Helen Schneider's Hospital for Women, Rabin Medical Center, Petach Tikva, Israel; Audrey Baron, Department of Respiratory and Intensive Care Medicine, Pulmonary Hypertension National Referral Centre, Hôpital Bicêtre, Le Kremlin Bicêtre, Paris, France; Ana Calinescu, Swiss Pediatric Liver Center, Department of Pediatrics, Gynecology, and Obstetrics, University of Geneva, Geneva, Switzerland; Vittoria Camelli, Pediatric Gastroenterology Unit, Regina Margherita Children's Hospital, AOU Città della Salute e della Scienza di Torino, Italy; Debalina Chakrabarti, Dept of Radiodiagnosis, Avadh Hospital and Heart Center, Lucknow 226005, India; Nicolas Foureur, Centre d'éthique clinique de l'AP-HP, Hôpital Cochin, Paris, France; Mehdi Gadiri, University of Geneva, Geneva, Switzerland; Solène Le Cam, Université Paris-Saclay, Faculté de Médecine, Le Kremlin-Bicêtre, France; AP-HP, Centre de Référence des maladies rares du foie de l'enfant, Service de radiologie pédiatrique diagnostique et interventionnelle, Hôpital Bicêtre, Le Kremlin-Bicêtre, France; Caroline Lemoine, Ann and Robert Lurie Children's Hospital of Chicago, Chicago, USA; Josefina Martinelli, Swiss Pediatric Liver Center, Department of Pediatrics, Gynecology, and Obstetrics, University of Geneva, Geneva, Switzerland; Periklis Makrythanasis, Medical School, National and Kapodistrian University of Athens, Athens, Greece; Jai Patel, Leeds Teaching Hospitals NHS Trust, Leeds, United Kingdom; Tudor Pop, 2nd Pediatric Clinic of the University of Medicine and Pharmacy Cluj-Napoca, Romania; Alexis Ricoeur, Unité de radiologie abdominale et interventionnelle, Département diagnostic, University Hospitals of Geneva, Geneva, Switzerland; Fabien Robert, INSERM UMR_S 999, Pulmonary Hypertension: Pathophysiology and Novel Therapies, Hôpital Marie Lannelongue, Le Plessis Robinson, France; Nathalie Rock, Swiss Pediatric Liver Center, Department of Pediatrics, Gynecology, and Obstetrics, University of Geneva, Geneva, Switzerland; Vanessa Steger, Department of Pediatrics, Gynecology, and Obstetrics, University of Geneva, Geneva, Switzerland; Frank van Steenbeek, University of Utrecht, Utrecht, Netherlands.

Supplementary data

Supplementary data to this article can be found online at https://doi.org/10.1016/j.jhepr.2023.100933.

References

Author names in bold designate shared co-first authorship

[1] Sokollik C, Bandsma RHJ, Gana JC, van den Heuvel M, Ling SC. Congenital portosystemic shunt: characterization of a multisystem disease. J Pediatr Gastroenterol Nutr 2013;56:675–681. https://doi.org/10.1097/MPG.0b013e31828b3750.

[2] Ohno T, Muneuchi J, Ihara K, Yuge T, Kanaya Y, Yamaki S, et al. Pulmonary hypertension in patients with congenital portosystemic venous shunt: a previously unrecognized association. Pediatrics 2008;121:e892–e899. https://doi.org/10.1542/peds.2006-3411.

[3] Franchi-Abella S, Branchereau S, Lambert V, Fabre M, Steimberg C, Losay J, et al. Complications of congenital portosystemic shunts in children: therapeutic options and outcomes. J Pediatr Gastroenterol Nutr 2010;51:322–330. https://doi.org/10.1097/MPG.0b013e3181d9cb92.

[4] Bernard O, Franchi-Abella S, Branchereau S, Pariente D, Gauthier F, Jacquemin E. Congenital portosystemic shunts in children: recognition, evaluation, and management. Semin Liver Dis 2012;32:273–287. https://doi.org/10.1055/s-0032-1329896.

[5] Baiges A, Turon F, Simón-Talero M, Tasayco S, Bueno J, Zekrini K, et al. Congenital extrahepatic portosystemic shunts (abernethy malformation): an international observational Study. Hepatology 2020;71:658–669. https://doi.org/10.1002/hep.30817.

[6] DiPaola F, Trout AT, Walther AE, Gupta A, Sheridan R, Campbell KM, et al. Congenital portosystemic shunts in children: associations, complications, and outcomes. Dig Dis Sci 2020;65:1239–1251. https://doi.org/10.1007/s10620-019-05834-w.

[7] Lambert V, Ladarre D, Fortas F, Durand P, Hervé P, Gonzales E, et al. Cardiovascular disorders in patients with congenital portosystemic shunts: 23 years of experience in a tertiary referral centre. Arch Cardiovasc Dis 2021;114:221–231. https://doi.org/10.1016/j.acvd.2020.10.003.

[8] Fahmy DM, Mitchell PD, Jonas MM. Presentation, management, and outcome of congenital portosystemic shunts in children: the Boston Children's hospital experience. J Pediatr Gastroenterol Nutr 2022;75:81. https://doi.org/10.1097/MPG.0000000000003450.

[9] Tyraskis A, Davenport M, Deganello A, Sellars M, De Vito C, Kane P, et al. Complications of congenital portosystemic shunts: liver tumors are affected by shunt severity, but pulmonary and neurocognitive associations are not. Hepatol Int 2022;16:918–925. https://doi.org/10.1007/s12072-022-10328-5.

[10] Uchida H, Sakamoto S, Kasahara M, Kudo H, Okajima H, Nio M, et al. Longterm outcome of liver transplantation for congenital extrahepatic portosystemic shunt. Liver Transpl 2021;27:236–247. https://doi.org/10.1002/lt.25805.

[11] Murray CP, Yoo S-J, Babyn PS. Congenital extrahepatic portosystemic shunts. Pediatr Radiol 2003;33:614–620. https://doi.org/10.1007/s00247-003-1002-x.

[12] Lautz TB, Tantemsapya N, Rowell E, Superina RA. Management and classification of type II congenital portosystemic shunts. J Pediatr Surg 2011;46:308–314. https://doi.org/10.1016/j.jpedsurg.2010.11.009.

[13] Gong Y, Zhu H, Chen J, Chen Q, Ji M, Pa M, et al. Congenital portosystemic shunts with and without gastrointestinal bleeding - case series. Pediatr Radiol 2015;45:1964–1971. https://doi.org/10.1007/s00247-015-3417-6.

[14] Sanada Y, Mizuta K, Niki T, Tashiro M, Hirata Y, Okada N, et al. Hepatocellular nodules resulting from congenital extrahepatic portosystemic shunts can differentiate into potentially malignant hepatocellular adenomas. J Hepatobiliary Pancreat Sci 2015;22:746–756. https://doi.org/10.1002/jhbp.277.

[15] Guérin F, Blanc T, Gauthier F, Abella SF, Branchereau S. Congenital portosystemic vascular malformations. Semin Pediatr Surg 2012;21:233–244. https://doi.org/10.1053/j.sempedsurg.2012.05.006.

[16] Lautz TB, Shah SA, Superina RA. Hepatoblastoma in children with congenital portosystemic shunts. J Pediatr Gastroenterol Nutr 2016;62:542–545. https://doi.org/10.1097/MPG.0000000000001012.

[17] Bahadori A, Kuhlmann B, Debray D, Franchi-Abella S, Wacker J, Beghetti M, et al. Presentation of congenital portosystemic shunts in children. Children 2022;9:243. https://doi.org/10.3390/children9020243.

[18] Vilgrain V, Paradis V, Van Wettere M, Valla D, Ronot M, Rautou P-E. Benign and malignant hepatocellular lesions in patients with vascular liver diseases. Abdom Radiol 2018;43:1968–1977. https://doi.org/10.1007/s00261-018-1502-7.

[19] Dioguardi Burgio M, Ronot M, Salvaggio G, Vilgrain V, Brancatelli G. Imaging of hepatic focal nodular hyperplasia: pictorial review and diagnostic strategy. Semin Ultrasound CT MR 2016;37:511–524. https://doi.org/10.1053/j.sult.2016.08.001.

[20] Sempoux C, Paradis V, Komuta M, Wee A, Calderaro J, Balabaud C, et al. Hepatocellular nodules expressing markers of hepatocellular adenomas in Budd-Chiari syndrome and other rare hepatic vascular disorders. J Hepatol 2015;63:1173–1180. https://doi.org/10.1016/j.jhep.2015.06.017.

[21] Sorkin T, Strautnieks S, Foskett P, Peddu P, Thompson RJ, Heaton N, et al. Multiple β-catenin mutations in hepatocellular lesions arising in Abernethy malformation. Hum Pathol 2016;53:153–158. https://doi.org/10.1016/j.humpath.2016.02.025.

[22] WHO classification of tumours: digestive system tumours. 5th edvol. 1. WHO Classification of Tumours Editorial Board; 2019.

[23] **Sempoux C**, **Gouw ASH**, Dunet V, Paradis V, Balabaud C, Bioulac-Sage P. Predictive patterns of glutamine synthetase immunohistochemical staining in CTNNB1-mutated hepatocellular adenomas. Am J Surg Pathol 2021;45:477–487. https://doi.org/10.1097/PAS.0000000000001675.

[24] Klompenhouwer AJ, Thomeer MGJ, Dinjens WNM, de Man RA, Ijzermans JNM, Doukas M. Phenotype or genotype: decision-making

dilemmas in hepatocellular adenoma. Hepatology 2019;70:1866–1868. https://doi.org/10.1002/hep.30812.

[25] McLin VA, Franchi Abella S, Debray D, Guérin F, Beghetti M, Savale L, et al. Congenital portosystemic shunts: current diagnosis and management. J Pediatr Gastroenterol Nutr 2019;68:615–622. https://doi.org/10.1097/MPG.0000000000002263.

[26] Moroz V, Morland B, Tiao G, Hiyama E, Kearns P, Wheatley K. The paediatric hepatic international tumour trial (PHITT): clinical trial design in rare disease. Trials 2015;16:P224. https://doi.org/10.1186/1745-6215-16-S2-P224.

[27] Humbert M, Kovacs G, Hoeper MM, Badagliacca R, Berger RMF, Brida M, et al. 2022 ESC/ERS Guidelines for the diagnosis and treatment of pulmonary hypertension. Eur Respir J 2022. https://doi.org/10.1183/13993003.00879-2022.

[28] Galiè N, Channick RN, Frantz RP, Grünig E, Jing ZC, Moiseeva O, et al. Risk stratification and medical therapy of pulmonary arterial hypertension. Eur Respir J 2019;53. https://doi.org/10.1183/13993003.01889-2018.

[29] Sitbon O, Bosch J, Cottreel E, Csonka D, de Groote P, Hoeper MM, et al. Macitentan for the treatment of portopulmonary hypertension (PORTICO): a multicentre, randomised, double-blind, placebo-controlled, phase 4 trial. Lancet Respir Med 2019;7:594–604. https://doi.org/10.1016/S2213-2600(19)30091-8.

[30] Joye R, Lador F, Aggoun Y, Farhat N, Wacker J, Wildhaber BE, et al. Outcome of paediatric portopulmonary hypertension in the modern management era: a case report of 6 patients. J Hepatol 2021;74:742–747. https://doi.org/10.1016/j.jhep.2020.11.039.

[31] Savale L, Guimas M, Ebstein N, Fertin M, Jevnikar M, Renard S, et al. Portopulmonary hypertension in the current era of pulmonary hypertension management. J Hepatol 2020;73:130–139. https://doi.org/10.1016/j.jhep.2020.02.021.

[32] Wu J, Lu Y, Zhao W, Shen J, Li F, Zhang H, et al. Clinical characteristics and therapeutic outcomes of pulmonary arterial hypertension secondary to congenital portosystemic shunts. Eur J Pediatr 2021;180:929–936. https://doi.org/10.1007/s00431-020-03817-y.

[33] Baumgartner H, De Backer J, Babu-Narayan SV, Budts W, Chessa M, Diller G-P, et al. 2020 ESC Guidelines for the management of adult congenital heart disease. Eur Heart J 2021;42:563–645. https://doi.org/10.1093/eurheartj/ehaa554.

[34] Rosenzweig EB, Abman SH, Adatia I, Beghetti M, Bonnet D, Haworth S, et al. Paediatric pulmonary arterial hypertension: updates on definition, classification, diagnostics and management. Eur Respir J 2019;53:1801916. https://doi.org/10.1183/13993003.01916-2018.

[35] Hoeper MM, Benza RL, Corris P, de Perrot M, Fadel E, Keogh AM, et al. Intensive care, right ventricular support and lung transplantation in patients with pulmonary hypertension. Eur Respir J 2019;53:1801906. https://doi.org/10.1183/13993003.01906-2018.

[36] Krowka MJ, Fallon MB, Kawut SM, Fuhrmann V, Heimbach JK, Ramsay MAE, et al. International liver transplant society practice guidelines: diagnosis and management of hepatopulmonary syndrome and portopulmonary hypertension. Transplantation 2016;100:1440–1452. https://doi.org/10.1097/TP.0000000000001229.

[37] DeMartino ES, Cartin-Ceba R, Findlay JY, Heimbach JK, Krowka MJ. Frequency and outcomes of patients with increased mean pulmonary artery pressure at the time of liver transplantation. Transplantation 2017;101:101–106. https://doi.org/10.1097/TP.0000000000001517.

[38] Khaderi S, Khan R, Safdar Z, Stribling R, Vierling JM, Goss JA, et al. Long-term follow-up of portopulmonary hypertension patients after liver transplantation. Liver Transpl 2014;20:724–727. https://doi.org/10.1002/lt.23870.

[39] Savale L, Sattler C, Coilly A, Conti F, Renard S, Francoz C, et al. Long-term outcome in liver transplantation candidates with portopulmonary hypertension. Hepatology 2017;65:1683–1692. https://doi.org/10.1002/hep.28990.

[40] Bas S, Guran T, Atay Z, Haliloglu B, Abalı S, Turan S, et al. Premature pubarche, hyperinsulinemia and hypothyroxinemia: novel manifestations of congenital portosystemic shunts (Abernethy malformation) in children. Horm Res Paediatr 2015;83:282–287. https://doi.org/10.1159/000369395.

[41] Rabah SA, Gowan IL, Pagnin M, Osman N, Richardson SJ. Thyroid hormone distributor proteins during development in vertebrates. Front Endocrinol (Lausanne) 2019;10:506. https://doi.org/10.3389/fendo.2019.00506.

[42] **Rhyu J**, **Yu R**. Newly discovered endocrine functions of the liver. World J Hepatol 2021;13:1611–1628. https://doi.org/10.4254/wjh.v13.i11.1611.

[43] McLin V, Franchi-Abella S, Debray D, Korff S, Casotti V, Colledan M, et al. FRI-436-Congenital porto-systemic shunts in children: preliminary results from the IRCPSS. J Hepatol 2019;70:e586. https://doi.org/10.1016/S0618-8278(19)31173-9.

[44] Francois B, Lachaux A, Gottrand F, De Smet S. Prenatally diagnosed congenital portosystemic shunts. J Matern Fetal Neonatal Med 2018;31:1364–1368. https://doi.org/10.1080/14767058.2017.1315093.

[45] Delle Chiaie L, Neuberger P, Von Kalle T. Congenital intrahepatic portosystemic shunt: prenatal diagnosis and possible influence on fetal growth. Ultrasound Obstet Gynecol 2008;32:233–235. https://doi.org/10.1002/uog.6116.

[46] Han BH, Park SB, Song MJ, Lee KS, Lee Y-H, Ko SY, et al. Congenital portosystemic shunts: prenatal manifestations with postnatal confirmation and follow-up. J Ultrasound Med 2013;32:45–52. https://doi.org/10.7863/jum.2013.32.1.45.

[47] Kim JY, Kim KA, Lee YK, Ko SY, Shin SM, Han BH. A clinical Study of congenital intrahepatic portosystemic shunt diagnosed in neonatal period. J Korean Soc Neonatol 2011;18:117–123. https://doi.org/10.5385/jksn.2011.18.1.117.

[48] Timpanaro T, Passanisi S, Sauna A, Trombatore C, Pennisi M, Petrillo G, et al. Congenital portosystemic shunt: our experience. Case Rep Pediatr 2015;2015:691618. https://doi.org/10.1155/2015/691618.

[49] Starzl TE, Putnam CW, Porter KA, Halgrimson CG, Corman J, Brown BI, et al. Portal diversion for the treatment of glycogen storage disease in humans. Ann Surg 1973;178:525–539. https://doi.org/10.1097/00000658-197310000-00015.

[50] Satoh M, Yokoya S, Hachiya Y, Hachiya M, Fujisawa T, Hoshino K, et al. Two hyperandrogenic adolescent girls with congenital portosystemic shunt. Eur J Pediatr 2001;160:307–311. https://doi.org/10.1007/s004310000539.

[51] Fuqua JS. Treatment and outcomes of precocious puberty: an update. J Clin Endocrinol Metab 2013;98:2198–2207. https://doi.org/10.1210/jc.2013-1024.

[52] Tripathi PR, Sen Sarma M, Yachha SK, Aggarwal A, Bhatia V, Kumar A, et al. Relative adrenal insufficiency in decompensated cirrhotic children: does it affect outcome? Am J Gastroenterol 2022;117:120–128. https://doi.org/10.14309/ajg.0000000000001486.

[53] **Bertino G**, **Privitera G**, Purrello F, Demma S, Crisafulli E, Spadaro L, et al. Emerging hepatic syndromes: pathophysiology, diagnosis and treatment. Intern Emerg Med 2016;11:905–916. https://doi.org/10.1007/s11739-016-1478-7.

[54] De Vito C, Tyraskis A, Davenport M, Thompson R, Heaton N, Quaglia A. Histopathology of livers in patients with congenital portosystemic shunts (Abernethy malformation): a case series of 22 patients. Virchows Arch 2019;474:47–57. https://doi.org/10.1007/s00428-018-2464-4.

[55] Dezsofi A, Baumann U, Dhawan A, Durmaz O, Fischler B, Hadzic N, et al. Liver biopsy in children: position paper of the ESPGHAN hepatology committee. J Pediatr Gastroenterol Nutr 2015;60:408. https://doi.org/10.1097/MPG.0000000000000632.

[56] Lemoine C, Nilsen A, Brandt K, Mohammad S, Melin-Aldana H, Superina R. Liver histopathology in patients with hepatic masses and the Abernethy malformation. J Pediatr Surg 2019;54:266–271. https://doi.org/10.1016/j.jpedsurg.2018.10.083.

[57] Lisovsky M, Konstas AA, Misdraji J. Congenital extrahepatic portosystemic shunts (abernethy malformation): a histopathologic evaluation. Am J Surg Pathol 2011;35:1381. https://doi.org/10.1097/PAS.0b013e3182230ce4.

[58] Roggen M, Cools B, Maleux G, Gewillig M. A custom-made percutaneous flow-restrictor to manage a symptomatic congenital porto-systemic shunt in an infant. Catheterization Cardiovasc Interventions 2018;92:92–95. https://doi.org/10.1002/ccd.27634.

[59] Bruckheimer E, Dagan T, Atar E, Schwartz M, Kachko L, Superina R, et al. Staged transcatheter treatment of portal hypoplasia and congenital portosystemic shunts in children. Cardiovasc Intervent Radiol 2013;36:1580–1585. https://doi.org/10.1007/s00270-013-0581-7.

[60] Facas J, Cruz M, Costa JF, Agostinho A, Donato P. Multistage closure of a congenital extrahepatic portosystemic shunt. CVIR Endovascular 2021;4:79. https://doi.org/10.1186/s42155-021-00267-x.

[61] Rao S, Karuppasamy K, Radhakrishnan K, Fagan TE. Restriction of congenital portosystemic shunt using the modified microvascular plug. Catheter Cardiovasc Interv 2021;98:1358–1362. https://doi.org/10.1002/ccd.29934.

[62] Ponce-Dorrego M-D, Hernández-Cabrero T, Garzón-Moll G. Endovascular treatment of congenital portosystemic shunt: a single-center prospective

Study. Pediatr Gastroenterol Hepatol Nutr 2022;25:147–162. https://doi.org/10.5223/pghn.2022.25.2.147.

[63] Blanc T, Guerin F, Franchi-Abella S, Jacquemin E, Pariente D, Soubrane O, et al. Congenital portosystemic shunts in children: a new anatomical classification correlated with surgical strategy. Ann Surg 2014;260:188–198. https://doi.org/10.1097/SLA.0000000000000266.

[64] Fahmy DM, Mitchell PD, Jonas MM. Presentation, management, and outcome of congenital portosystemic shunts in children: the Boston Children's hospital experience. J Pediatr Gastroenterol Nutr 2022;75:81–87. https://doi.org/10.1097/MPG.0000000000003450.

[65] Franchi-Abella S, Gonzales E, Ackermann O, Branchereau S, Pariente D, Guérin F, et al. Congenital portosystemic shunts: diagnosis and treatment. Abdom Radiol (Ny) 2018;43:2023–2036. https://doi.org/10.1007/s00261-018-1619-8.

[66] McLin VA, Franchi-Abella S, Debray D, Korff S, Casotti V, Colledan M, et al. Intrahepatic and extrahepatic congenital portosystemic shunts differ in in clinical presentation and outcomes in children. Hepatology 2019. November, Boston, USA: abstract 14181.

[67] **Cytter-Kuint R**, **Slae M**, Kvyat K, Shteyer E. Characterization and natural history of congenital intrahepatic portosystemic shunts. Eur J Pediatr 2021;180:1733–1737. https://doi.org/10.1007/s00431-021-03949-9.

[68] Eroglu Y, Donaldson J, Sorensen LG, Vogelzang RL, Melin-Aldana H, Andersen J, et al. Improved neurocognitive function after radiologic closure of congenital portosystemic shunts. J Pediatr Gastroenterol Nutr 2004;39:410–417. https://doi.org/10.1097/00005176-200410000-00019.

[69] Achiron R, Kivilevitch Z. Fetal umbilical-portal-systemic venous shunt: in-utero classification and clinical significance. Ultrasound Obstet Gynecol 2016;47:739–747. https://doi.org/10.1002/uog.14906.

[70] Achiron R, Kassif E, Kivilevitch Z. Fetal intrahepatic Umbilical-Porto-Systemic venous shunts (IHUPSVS): in-utero anatomic classification. Eur J Obstet Gynecol Reprod Biol 2022;276:179–184. https://doi.org/10.1016/j.ejogrb.2022.07.022.

[71] **Kivilevitch Z**, **Kassif E**, Gilboa Y, Weisbuch T, Achiron R. The intra-hepatic umbilical-Porto-systemic venous shunt and fetal growth. Prenat Diagn 2021;41:457–464. https://doi.org/10.1002/pd.5882.

Journal of Hepatology, Volume *6*

Supplemental information

Expert management of congenital portosystemic shunts and their complications

Valérie Anne McLin, Stéphanie Franchi-Abella, Timothée Brütsch, Atessa Bahadori, Valeria Casotti, Jean de Ville de Goyet, Grégoire Dumery, Emmanuel Gonzales, Florent Guérin, Sebastien Hascoet, Nigel Heaton, Béatrice Kuhlmann, Frédéric Lador, Virginie Lambert, Paolo Marra, Aurélie Plessier, Alberto Quaglia, Anne-Laure Rougemont, Laurent Savale, Moinak Sen Sarma, Olivier Sitbon, Riccardo Antonio Superina, Hajime Uchida, Mirjam van Albada, Hubert Petrus Johannes van der Doef, Valérie Vilgrain, Julie Wacker, Nitash Zwaveling, Dominique Debray, and Barbara Elisabeth Wildhaber

Expert management of congenital portosystemic shunts and their complications

Paolo Marra, Stephanie Franchi-Abella, Timothée Brütsch, Valérie McLin

Table of contents

How to perform an occlusion test…………………………………………………………..……..2

Supplementary references…………………………………………………………………….. 2

How to perform an occlusion test

The occlusion test aims to simulate the portosystemic shunt closure: to allow the opacification of a hypoplastic and/or ectopic portal system, if present, and to depict its anatomy; to monitor the increase of portal pressure and to rule out the development of portal hypertension or the opening of other neglected portosystemic communications; to plan the radiological embolization of the shunt. To be effective, the occlusion test must avoid the inadvertent closure of ectopic portal vessels that may originate close to the portosystemic communication, while completely occluding the shunt lumen [1]. The procedure can be performed via the transfemoral and/or the transjugular route under sedation or general anaesthesia, according to the clinical context and resources. Since extrahepatic end-to-side and side-to-side shunts may be very large, dedicated occlusion balloons are preferrable. For example, the Equalizer balloon catheter is currently available on-the-market. Large-caliber aortic or valvuloplasty balloons generally have an ellipsoid shape, which may hinder the correct execution of the test. The balloon should be inflated within the inferior vena cava or the systemic afferent vein at the shunt orifice. If the tip of the balloon catheter is navigated within the shunt, the portal pressure can be determined through the main lumen of the balloon catheter after removing the guidewire. If the tip is in the vena cava lumen, a side catheterization of the spleno-mesenteric axis must be performed. It is important not to perform the occlusion of the vena cava at the level of the hepatic vein confluence, to avoid overestimating the portal pressure due to hepatic outflow obstruction. Intrahepatic or extrahepatic shunts originating distant from the spleno-mesenteric confluence may be occluded more selectively, with widely available smaller occlusion balloon catheters. There is no consensus about the duration of the occlusion test. Given that prolonged occlusion of large shunts may cause hemodynamic collapse or thrombosis, it is reasonable to limit occlusion duration just to the time required for wedge portal pressure measurement and portal venography execution. In such circumstances heparin prophylaxis is not recommended. No consensus exists also regarding a cut-off for portal pressure value allowing the choice between one-stage and two-stage shunt closure: an absolute wedge portal pressure >30 mmHg and/or a >20 mmHg gradient between free and wedge portal pressure are commonly adopted criteria to promote a two-stage closure [2,3].

Supplementary references

[1] Franchi-Abella S, Gonzales E, Ackermann O, Branchereau S, Pariente D, Guérin F, et al. Congenital portosystemic shunts: diagnosis and treatment. Abdom Radiol (NY) 2018;43:2023–36. https://doi.org/10.1007/s00261-018-1619-8.

[2] Matsuura T, Takahashi Y, Yanagi Y, Yoshimaru K, Yamamura K, Morihana E, et al. Surgical strategy according to the anatomical types of congenital portosystemic shunts in children. Journal of Pediatric Surgery 2016;51:2099–104. https://doi.org/10.1016/j.jpedsurg.2016.09.046.

[3] Rajeswaran S, Johnston A, Green J, Riaz A, Thornburg B, Mouli S, et al. Abernethy Malformations: Evaluation and Management of Congenital Portosystemic Shunts. J Vasc Interv Radiol 2020;31:788–94. https://doi.org/10.1016/j.jvir.2019.08.007.
